# Herpes simplex virus and kallikrein-related peptidases – a functional connection in Alzheimer‘s disease

**DOI:** 10.1186/s13195-026-02204-3

**Published:** 2026-09-24

**Authors:** Oliver Goldhardt, Cinthia Mella Robles, Matthias Eberl, Tobias Dreyer, Antoninus Soosaipillai, Eleftherios P. Diamandis, Josef Priller, Dieter Hoffmann, Jochen M. Wettengel, Hianara Aracelly Bustamante, Paula Salazar, Markus Thaler, Carola Otth, Ingrid Pamela Ehrenfeld Slater, Timo Grimmer

**Affiliations:** 1 Department of Neurology, TUM University Hospital, TUM School of Medicine and Health, Technical University of Munich (TUM), Ismaninger Str. 22, Munich, 81675 Germany; 2 Laboratory of Molecular Virology, Institute of Clinical Microbiology, Faculty of Medicine, University Austral de Chile, Los Laureles S/N, Isla Teja, Valdivia, 5110566 Chile; 3 Department of Obstetrics & Gynecology, TUM University Hospital, TUM School of Medicine and Health, Technical University of Munich (TUM), Ismaninger Str. 22, Munich, 81675 Germany; 4 Lunenfeld-Tanenbaum Research Institute, Sinai Health System, 60 Murray Street, Toronto, ON M5T 3L9 Canada; 5 Department of Laboratory Medicine and Pathobiology, Faculty of Medicine, University of Toronto, Medical Science Building, 1 King’s College Circle, Toronto, ON M5S 1A8 Canada; 6 Laboratory of Molecular Psychiatry and DZNE, Charitéplatz 1, Berlin, 10117 Germany; 7 University of Edinburgh and UK DRI, 49 Little France Cres, Edinburgh, EH16 4TJ UK; 8 Institute of Virology, TUM School of Medicine and Health, Helmholtz Munich / Technical University of Munich (TUM), Trogerstr. 30, Munich, 81675 Germany; 9 Department of Laboratory Medicine, TUM University Hospital, TUM School of Medicine and Health, Technical University of Munich (TUM), Ismaninger Str. 22, Munich, 81675 Germany; 10 Laboratory of Cellular Pathology, Institute of Anatomy, Histology and Pathology, Faculty of Medicine, University Austral de Chile, Los Laureles S/N, Isla Teja, Valdivia, 5110566 Chile; 11 Department of Psychiatry and Psychotherapy, TUM University Hospital, TUM School of Medicine and Health, Technical University of Munich (TUM), Ismaninger Str. 22, Munich, 81675 Germany

**Keywords:** Herpes simplex virus type 1, Alpha-herpesvirinae, HSV1, KLK6, Kallikrein-related peptidase, Alzheimer’s disease, Total tau, Phospho tau, Soluble amyloid

## Abstract

**Background:**

The viral hypothesis of Alzheimer’s disease (AD) proposes that neurotropic pathogens may interact with amyloid and tau pathology, but the evidence remains controversial. We investigated whether cerebrospinal fluid (CSF) kallikrein-related peptidase 6 (KLK6) is associated with AD biomarkers and with an intrathecal anti-HSV immune response in patients with AD, and whether HSV-1 infection alters KLK6 expression in cell culture models.

**Methods:**

CSF KLKs and AD biomarkers were analyzed in patients with AD. HSV-1 serostatus and anti-HSV-1/2 CSF-to-serum antibody index (HSV-AI) were used as markers of previous or ongoing intrathecal anti-HSV immune response. In vitro, HSV-1 infection and KLK6 knockdown were examined in human cell models.

**Results:**

CSF-KLK6 correlated with tau and amyloid biomarkers. In HSV-1-seropositive patients, HSV-AI was associated with CSF-KLK6, although the effect size was modest and the association was strongly context-dependent on the Aβ42/40 ratio. In vitro, productive HSV-1 infection induced and stabilized KLK6 protein expression, and KLK6 knockdown reduced HSV-1 protein expression and infectious progeny in HaCaT cells. Conclusions: KLK6 is associated with AD biomarkers and with an intrathecal anti-HSV immune response in a subset of patients with AD. The in vitro data support a functional relationship between HSV-1 infection and KLK6, but the clinical findings are associative and do not demonstrate ongoing CNS HSV-1 replication or causality. These findings warrant independent validation. However, this research provides further evidence for a role of HSV-1 in the pathophysiology of AD.

**Graphical Abstract:**

**Figure:**
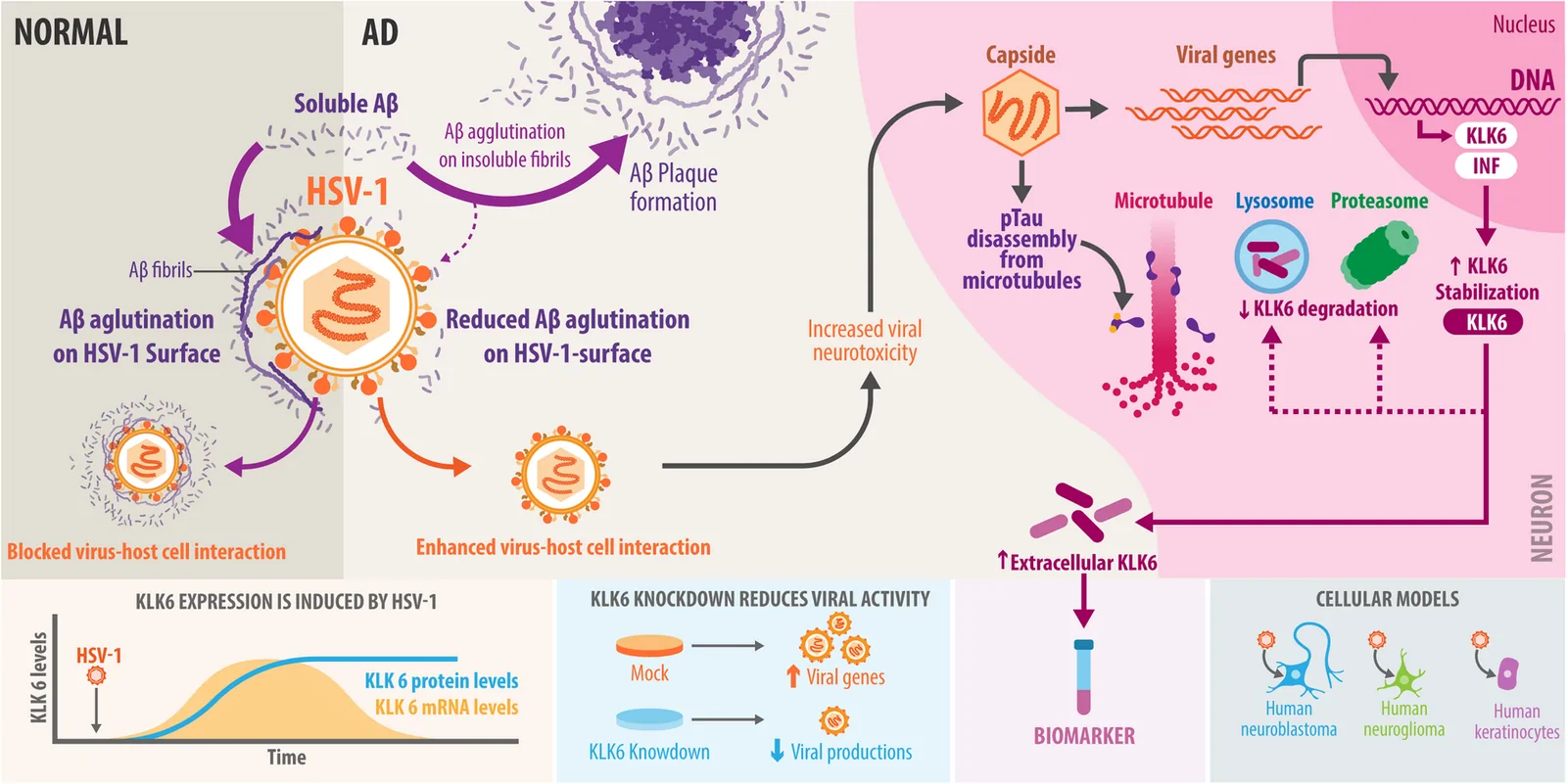

**Supplementary Information:**

The online version contains supplementary material available at https://doi.org/10.1186/s13195-026-02204-3.

## Background

Alzheimer’s disease (AD) is histopathologically defined by intraneuronal fibrillary tangles of hyperphosphorylated tau and deposition of extracellular fibrils of beta-amyloid (Aβ) in the brain [1]. The likelihood of Aβ fibril accumulation increases with an elevated Aβ concentration in the brain due to Aβ overproduction in familial AD [2] or impaired Aβ clearance in sporadic AD [3, 4]. Aβ oligomers impair the lysosomal function, exert neurotoxic effects and cause direct inflammatory stimuli as danger-associated molecular patterns (DAMPs) [5]. A consequence of insoluble Aβ fibril accumulation is a decreasing soluble Aβ concentration in the brain, due to the equilibrium between monomers, oligomers and fibrils. Among different Aβ cleavage products, Aβ42 shows the highest fibrillary potential [6]. In the cerebrospinal fluid (CSF), a decreased ratio of Aβ42 to Aβ40 (Aβ42/Aβ40 ratio), and elevated phospho Tau (pTau) and total Tau (tTau) levels correlate with AD pathology and are used for diagnostics [7]. So far, the causative relation between Aβ and tau pathology remains elusive and is a subject of current debate [8]. Compared to the regional progression of Aβ fibril accumulation in the brain, the spread of tau pathology in the brain correlates far more strongly with the clinical symptoms (i.e. mnestic deficits) and the extent of neurodegeneration in AD [9]. In addition, the causes of AD appear to be multifactorial, and CSF AD biomarkers used in differential diagnostics provide only limited predictive value for disease progression, especially in the early stages of AD.

The search for and validation of biomarkers indicating additional aspects of early pathophysiology in AD to improve diagnostic accuracy is of great interest for disease-modifying therapy. In recent decades, the physiological function of Aβ has been increasingly studied as Aβ is abundant in the brain and has persisted by evolutionary selection. Besides linkage of various Aβ species with memory consolidation and synaptic plasticity, especially Aβ42 has been found to exhibit inhibitory effects against various neurotrophic pathogens [10]. This is also true for enveloped human-pathogenic herpesviruses consisting of 9 members which are divided into three different subfamilies: *alphaherpesvirinae* with i.e. the herpes simplex virus type 1 (HSV-1) and the varicella zoster virus (VZV), *betaherpesvirinae* with i.e. the cytomegalovirus (CMV) and *gammaherpesvirinae*. About 80% of humans are infected by HSV-1, mostly with mild or subclinical manifestations [11]. After initial infection of a host cell, herpesviruses have the ability to enter a stage of life-long latency and can reactivate after certain stimuli. Typically, HSV-1 establishes latency in the trigeminal ganglion cells, from where reactivation can lead to infectious dermal efflorescences. However, via interneuronal fiber connections, HSV-1 can also reach other brain regions, such as the temporal lobe including the hippocampus [12, 13], which is dominantly affected in AD. Therefore, HSV-1 reactivation is suspected to pose a risk for neurodegeneration. Aβ can form oligomers that have been shown to bind several glycoproteins of HSV-1 envelope [14], which can prevent the virus from interacting with host cells and reduce its viral replication ability in the brain [15, 16].

HSV-1 infection and reactivation also causes hyperphosphorylation of tau in a neuronal cell culture and AD animal model [12, 13, 17] by induction of viral and human kinases [13, 17], leading to hyperphosphorylation of various intracellular molecules including tau [18, 19]. In a murine AD model of recurrent HSV-1 reactivation, Aβ and tau pathology were significantly triggered [12]. The significant association of CSF pTau and CSF tTau with the intrathecal anti-HSV-1 immune response, assessed by the CSF-to-serum anti-HSV-1/2 IgG antibody index (HSV-AI) as a biomarker of recurrent antiviral humoral immune activation compatible with previous or intermittent HSV antigenic stimulation (likely reflecting viral reactivation in HSV-1-seropositive individuals), has recently been confirmed in patients with AD [20]. Consistent with the virostatic effect of soluble Aβ, this association was most pronounced in patients with the lowest Aβ42/40 ratios. These findings suggest that patients with AD may become increasingly susceptible to HSV-1-associated neurotoxicity as soluble Aβ levels decline [20]. Moreover, neurotropic pathogens such as HSV-1 have been proposed to act as potential Aβ seeding agents decades before the clinical onset of AD.

However, the evidence linking herpesviruses to AD remains heterogeneous. Transcriptomic analyses by Readhead et al. identified an association of HHV-6 A and HHV-7, rather than HSV-1, with molecular networks related to AD pathology [21]. However, these findings could not be consistently reproduced in subsequent re-analyses and independent cohorts, which questioned the robustness of the reported viral sequence enrichment [22, 23]. Likewise, previous studies investigating HSV DNA in post-mortem brain tissue have yielded inconsistent results, ranging from frequent detection to very low prevalence, likely reflecting methodological and cohort-related differences [24, 25]. Rather than detecting viral nucleic acids in post-mortem brain tissue, we investigated the intrathecal HSV-specific immune response as a functional marker of recurrent CNS antigen exposure in living patients with biomarker-confirmed AD. This approach also supports the hypothesis that HSV-related mechanisms may be relevant only in a subset of patients with AD. Accordingly, our hypothesis is not that HSV-1 is a primary cause of Alzheimer’s disease, but rather that increasing amyloid pathology may increase susceptibility to herpesvirus-associated immune responses in susceptible individuals.

Independent of pathogens, AD is associated with a high inflammatory status in the brain including cell types like microglia, glia cells and neurons. Aβ triggers the inflammatory immune cascade [26]. Numerous inflammatory signals are released and reach the blood and CSF. The kallikrein-related peptidases (KLKs) comprise a homologous family of 15 secreted serine proteases that participate in inflammatory and extracellular proteolytic processes. KLKs show a trypsin- or chymotrypsin-like activity and are secreted into and activated in the extracellular space where they are activated [27]. In the brain, among others, KLK6-8 and 10 are expressed by various cell types, and have been suggested as AD biomarker candidates [28]. We have shown in patients with biomarker-confirmed AD that at least CSF-KLK6 and CSF-KLK10 proteins, but not CSF-KLK8^3^, are significantly increased compared with cognitively healthy controls and are correlated with AD biomarkers [3, 29].

KLK protein expression and function have been associated with viral infectivity and inflammatory response to various viruses, such as influenzavirus, papillomavirus and members of the *orthoherpesviridae* family [30]. In human keratinocytes, alphaherpesvirus infection is correlated with the induction of KLK5-8, KLK10 and KLK12. For instance, KLK expression is induced by HSV-1 triggered phosphorylation of transcription factor sp1 [31, 32] and VZV infection [33]. In VZV-infected keratinocytes, virus-induced KLK6 protein expression enhances viral infectivity, likely through downstream effects such as degradation of cytokeratin 10, which may facilitate VZV propagation [33].

As CSF-KLK6 [3] and intrathecal antibody synthesis against HSV-1/2 [20] correlate significantly with CSF-pTau181 in AD, we assume that CSF-KLK6 itself might be a biomarker for intrathecal anti-HSV immune response in AD. KLK6 can elucidate the pathophysiological connection between herpesviruses and AD. Furthermore, KLK6 has the potential to indicate very early virus-mediated alterations in the brain leading to AD. In this study, we also investigate whether KLK6 is physiologically connected to HSV-1 replication and correlates with productive infection of HSV-1. In order to delineate the specificity of the effects concerning KLK6 and HSV-1, also other KLKs as well as CMV were tested.

## Methods

This retrospective, monocentric clinical study with a small longitudinal subset of patients was conducted at a university-based outpatient memory clinic to investigate whether the biomarker KLK6 is suitable for indicating HSV-1–associated alterations in CSF biomarkers of Alzheimer’s disease (AD). In addition, the mechanistic role of KLK6 was examined in various in vitro cell models.

### Patient samples

114 CSF samples were available from our previously published cohort 117 patients at early stages of AD [20]. HSV-1 and CMV serostatus were determined. Intrathecal antibody indices were subsequently determined only in the corresponding seropositive individuals for each virus (see below). Consequently, the HSV- and CMV-seropositive subgroups partially overlapped, as some patients were seropositive for both viruses. For 80 anti-HSV-1-IgG seropositive patients, anti-HSV-1/2-IgG-CSF to serum antibody index (HSV-AI) was available. 24 patients were anti-HSV-1-IgG negative. In 10 cases HSV-AI could not be determined. For 37 anti-CMV-IgG seropositive patients anti-CMV-IgG-CSF to serum antibody index (CMV-AI) was available.

Study participants had been referred for the evaluation of cognitive impairment by standardized diagnostic procedure with a detailed somatic, neurologic and psychiatric examination, as well as standardized neuropsychological testing, including the Mini-Mental State examination (MMSE), and clinical severity was estimated by the global score of Clinical Dementia Rating scale (CDR). Diagnosis of AD, i.e. mild cognitive impairment, mild or moderate dementia due to probable AD, was made in accordance with the standard diagnostic criteria at time of enrollment ([1, 34]. Information on relevant systemic comorbidities and concomitant medication was obtained from the medical records. No patient received anti-amyloid antibody therapy, immunosuppressive treatment, or anticoagulant medication. The majority of cerebrospinal fluid (CSF) samples were obtained either during the diagnostic work-up or as part of screening procedures for prospective clinical trials, which applied strict eligibility criteria regarding systemic comorbidities and concomitant medication. Consequently, the study cohort was largely free of severe systemic illness or treatments expected to substantially influence immune function. The most common concomitant medications comprised antihypertensive agents, lipid-lowering drugs, antidiabetic medication, and acetylcholinesterase inhibitors. In the longitudinal subgroup, the first lumbar puncture was performed during the diagnostic evaluation, whereas the second lumbar puncture was obtained during clinical trial screening.

For longitudinal analyses, of the cross-sectional cohort, 18 patients had been selected with longitudinal lumbar puncture (LP) with a mean interval between both LPs of 0.99 ± 0.578 (range 0.3-2.0), of which 17 were HSV-1-seropositive. Mean alteration rates of all CSF AD biomarkers were calculated per year.

The present study focuses exclusively on patients with AD. Although a non-AD control cohort was included in our previous study (Goldhardt et al., 2023), no remaining CSF samples from these individuals were available for KLK6 measurements and they therefore could not be included in the present analyses.

### CSF ELISA (KLK6, KLK7, KLK8, KLK10)

Methods have been described elsewhere [3]. Immunofluorometric ELISA techniques have been described elsewhere [35–37]. KLKs were measured blinded in duplicates. The inter-assay CVs were less than 10% for all assays. For CSF, recovery percentages for all KLKs were between 90 and 110%. The antibody against KLK6 does not exhibit cross-reactivity with a series of different KLKs, KLK2-3, KLK8, KLK10-11, KLK [13, 21], even when the cross-reactants were tested at concentrations of 1000 µg/L [38]. The antibody against KLK8 displayed no cross-reactivity against members of the human kallikrein family [36]. Finally, the antibody against KLK10 showed no cross-reactivity with other homologous kallikrein proteins, such as KLK3, KLK2, and KLK6, as well [37]. The method for quantifying KLK7 is similar to the one published for KLK8, but specific polyclonal mouse and rabbit antibodies against KLK7 were used [31].

### HSV ELISAs

Methods have been described elsewhere [20]. Intrathecal antibody indices were calculated according to the established Reiber method, thereby correcting for individual blood–CSF barrier function through incorporation of the CSF to serum albumin quotient (Q_alb_) and the corresponding hyperbolic discrimination limit. Alegria 8-well-microstrips anti-HSV-1/2-IgG-CSF (ORG905GL), anti-HSV-1-IgG-Serum (ORG903G), and anti-HSV-1-IgM (ORG903MX), which have been approved for European conformity (CE), were purchased from Orgentec Diagnostika GmbH (Mainz, Germany). ELISAs were performed according to manufacturer’s instructions (www.orgentec.com), and test strips were measured photometrically at 650 nm on an Alegria instrument (Orgentec Diagnostika GmbH) using Sensotronic Memorized Calibration SMC. ORG903G was used exclusively to determine HSV-1 seropositivity (HSV+), as no cutoff for HSV-1 seropositivity is defined for ORG905L. The detection limit of serum anti-HSV-1-IgG is 5.9 U/ml when measured linearly. HSV-1-seropositivity was interpreted by anti-HSV-1-IgG serum antibodies according to the manufacturer’s information as negative if < 20 U/ml, borderline if 20 to 25 U/ml, and positive if > 25 U/ml for HSV-1 infection or reactivation, respectively. The detection limit of anti-HSV-1/2-IgG-CSF was 10 U/ml. CSF and serum were diluted according to the protocol, and protein concentrations were assessed, including the ratio of albumin serum-to-CSF (Q_alb_) and the ratio of nonspecific total IgG (Q_IgG_). No patient showed a polyspecific immune response. The anti-HSV-1/2-IgG CSF-to-serum antibody index (HSV-AI) was calculated by dividing the specific anti-HSV-1/2-IgG CSF-to-serum titer (Q_spec_ = (IgG_spec_ CSF x CSF dilution factor) / (IgG_spec_ serum x serum dilution factor)) by the total IgG CSF-to-serum titer (Q_IgG_ = total IgG CSF / total IgG serum) with reference to the upper discrimination line (Q_lim_) of the reference range of the blood-derived protein fraction in CSF [20]. HSV-AI values range from 0.5 to less than 1.5 was interpreted as normal and ≥ 1.5 as intrathecal synthesis of specific anti-HSV-1/2 antibodies.

Q_alb_ did not correlate significantly with HSV-AI (Spearman ρ = 0.016, *p* = 0.884; Kolmogorow-Smirnov (KS) test for Q_alb_*p* < 0.001) or with total Q_IgG_ (Spearman ρ= -0.002, *p* = 0.989; KS test for Q_IgG_*p* < 0.001). In addition, marked blood–CSF barrier dysfunction was uncommon in the present cohort (only in one case) and is therefore unlikely to have substantially influenced other CSF measurements, including KLK6. KLK6 furthermore did not correlate with Q_IgG_ nor with Q_alb_ [3].

### CMV ELISA

Methods have been described elsewhere [20]. Anti-CMV IgG antibodies in CSF and serum were measured in duplicate by ELISA (Abnova, KA1452) and repeated in adjusted dilutions if out of range. CSF was diluted at 1:2 and serum 1:404. A standard curve with a polynomic (four-parameter) function was calculated using SPSS. According to the published specifications of the ELISA’s protocol, semiquantitative positivity index on predetermined standard was used for the cutoff value. Sera that have absorbance value ≥ 90% cutoff were considered positive. Anti-CMV-IgG serum titers in AU/ml were calculated in CMV-seropositive patients and NCs. CSF-to-serum anti-CMV-IgG antibody indices (CMV-AI) were calculated similarly to HSV-AI specifications. Only valid values ≥ 0.5 were used for analyses, and values ≥ 1.5 indicated intrathecal antibody production.

### CSF ELISA (Aβ42, Aβ40, phosphorylated Tau181, total Tau)

CSF sampling and methods have been described elsewhere [20]. CSF-Aβ42 and CSF-Aβ40 were measured in triplicate, and CSF-pTau181, and CSF-tTau were measured in duplicate using commercially available ELISAs (IBL, amyloid beta [1–42] IBL RE 59661 and Amyloid beta [1–40] RE 59651; Fujirebio, Innotest P-TAU 81574 and hTAU Ag 81572). The coefficients of variation (CVs) were less than 6% for CSF-Aβ42 and CSF-Aβ40 and less than 3% for CSF-pTau181 and CSF-tTau. Aβ42/Aβ40 ratio was calculated.

### Chemical reagents and antibodies

The HALT protease and phosphatase inhibitor cocktail was purchased from Thermo Fisher Scientific (Waltham, MA, USA) and RIPA Lysis Buffer, Dimethyl sulfoxide (DMSO; CAS 67-68-5), Z-Leu-Leu-Leu-al (MG132; CAS 133407-82-6), and Bafilomycin A1 (Baf A1; CAS 88899-55-2) were purchased from Sigma Aldrich (St. Louis, MO, USA). The following antibodies were used: mouse anti-β-ACTIN (MA5-15739) and mouse anti-p53 (MA5-12557) were acquired from Thermo Fisher Scientific; mouse anti-ICP8 (sc-53329) and mouse anti-KRT10 (sc-23877) were acquired from Santa Cruz Biotechnology (Dallas, TX, USA), rabbit anti-HSV-1 total (ab9533), mouse anti-ICP0 (ab6513), mouse anti-ICP5 (ab6508), mouse anti-ICP27 (ab31631), rabbit anti-KLK1 (ab28289), rabbit anti-KLK5 (ab28565), rabbit anti-KLK6 (ab28301), rabbit anti-KLK7 (ab2000745), and mouse anti-KLK10 (ab55623) were acquired from Abcam, Inc. (Cambridge, UK), and rabbit anti-KLK12 (9604217) was acquired from MyBioSource, Inc. (San Diego, CA, USA).

### HSV-1 propagation and TCID50 titration

HSV-1 strain F was kindly provided by Dr. Bernard Roizman (The Marjorie B. Kovler Viral Oncology Laboratories, The University of Chicago, Chicago, IL, USA). The virus stocks were propagated, titrated in Vero cells by TCID50 assay 50. HSV-1 titer was measured through End-Point Dilution Assay known as TCID50 (Tissue Cultured Infective Dose), where Vero cells were plated in a 96-well plate and grown until reaching confluence. The monolayer cells were incubated for 1 h at 37 °C with serial 10-fold diluted HSV-1 suspensions in DMEM culture medium. The medium was aspirated and fresh DMEM containing 5% FBS was added, incubating the plates at 37 °C. Estimation of the endpoints was made at 96 h for visualization and registry of the cytopathic effect for each dilution. The viral dose infective necessary to produce a cytopathic effect in 50% of the infected cells was calculated as TCID50/mL according to Spearman–Kärber method. The titration was performed in three biological replicates with eight technical replicates for each dilution and condition. The final results were expressed as log10TCID50/ml.

### In vitro HSV-1 infection model

Cell cultures of H4 (human neuroglioma ATCC^®^ HTB-148TM; Manassas, VA, USA), HaCaT supplied by Dr N. Fusenig (DKFZ, Heidelberg, Germany) and SH-SY5Y (Neuroblastoma humano ATCC^®^ CRL-2266TM; Manassas, VA, USA) were maintained in monolayer in Dulbecco’s Modified Eagle Medium (DMEM) 1X (Life Technologies, Inc), supplemented with 10% fetal bovine serum (FBS) (Life Technologies, Inc) and 1% (v/v) antibiotics penicillin-streptomycin (Thermo Scientific™, Waltham, MA, USA) (100 units/ml) and incubated at 5% CO2 and 37 °C, in relative humidity of 95%. Cell cultures were infected with multiplicity of infection (MOI) = 10 (multiplicity of infection 10:1 viral particles/cell) for 1 h (viral adsorption stage). As an infection control or Mock (M), only the virus vehicle was used as an inoculum without containing virus (DMEM 1 × 0% FBS medium) and then the viral adsorption procedure was followed. After this, the medium was changed for fresh medium without FBS, and incubated for different times, according to the infection kinetics M, 1, 4, 8, 18 and 24 hpi, taking as time 0 post infection, the change of medium after the 1 h incubation of adsorption, both for the Mock, and for all times of the kinetics. For the treatment with drugs (CHX, BafA1, and MG132) and silencing (siNT and siKLK6) the same infection protocol was performed but using only the Mock and the 18 hpi. All processes involving the handling of samples contaminated with live viruses were placed in autoclavable bags and autoclaved prior to disposal; procedures that were carried out considering the biosafety measures provided for in the “Biosafety Standards Manual”, published by CONICYT in 1994. For protein stability HaCaT cells at 90% confluence were infected with HSV-1 (F strain) and treated with cycloheximide (100 µg/ml) at 10 hpi to inhibit protein synthesis. Cells were collected at 1, 2, 4, and 8 h post-treatment in a final point of infection at 18 hpi, for Western blot analysis.

### KLK6 silencing

KLK6 silencing was performed by transfecting KLK6 siRNA (siKLK6) acquired from Santa Cruz Biotechnology (sc-41532) into HaCaT cells. SiNT acquired from Ambion Inc. (AM4611) was used as negative control to determine baseline cellular response to siRNA delivery. Cells were seeded at approximately 30% confluence to initiate transfection. Hours prior to transfection, the propagation culture medium was replaced with DMEM 1X/Normocin 10% FBS. At the time of transfection, two mixes were prepared: (1) Mix 1: siRNA (100 µM)/Opti-MEM and (2) Mix 3: Lipofectamine RNAiMax/Opti-MEM, which were allowed to stand for 5 min, separately at room temperature. Afterwards, both mixes were mixed and allowed to stand for half an hour at room temperature, this mixture was added dropwise to each well with cells and kept at 37 °C for 24 h, reaching a final concentration of 100 nM in the culture. For the wells with double siRNA transfection, either siNT or siKLK6, the same procedure was repeated and maintained for 24 h more, under the same conditions (Transfection time: 48 h). Those wells with only one shot were returned to the propagation medium. After the transfection time, infection with HSV-1 was performed as described above, for 18 hpi. This experiment was intended for protein extraction and TCID50 assays.

### Overexpression of KLK6

The KLK6 vector DU57546, pCMV SPORT6 kindly donated by Dr. Alejandro Rojas (University Austral de Chile) and was used to overexpress KLK6 into H4 cells as a positive control, using Lipofectamine 3000 reagent (Invitrogen). H4 cells were grown on 12-well culture plates until reaching a confluence of 65–70%. At the time of transfection, cells were washed with 1X DPBS and the propagation culture medium was replaced with 1X DMEM/Normocin 10% FBS. At the time of transfection, two mixes were prepared: (1) Mix 1: DNA (4 µg)/Opti-MEM and (2) Mix 3: Lipofectamine 3000/Opti-MEM. Both mixes were mixed and left to rest for half an hour at room temperature, then this mixture was added dropwise to each well with cells and kept at 37 °C for 16 h.

### RT-qPCR assay

RNA was extracted from the HaCaT cell line according to M-8-18 hpi kinetics, using a total RNA extraction kit using the Direct-zolTM RNA extraction kit, RNA MiniPrep Plus (OMEGA bio-tek) according to the manufacturer’s instructions. RNA was quantified by spectrophotometry with Maestronano (MaestroGen). cDNA synthesis was performed from 2ug of RNA in a final reaction volume of 20ul with M-MLV Reverse Transcriptase (Promega), using random primers. The cDNA was diluted to a final concentration of 20ng/ul, and 10ng total were loaded per reaction. The RT-qPCR reaction was carried out with PowerUp™ SYBR^®^ Green Master Mix (Applied Biosystems) in a final volume of 20ul, in the CFX96 thermal cycler (BioRad), using a concentration of 0.3 μm of each primer in a 96-well plate (BioRad). The thermal cycling conditions were as follows: 50 °C for 2 min, 95 °C for 2 min, followed by 42 cycles of 95 °C for 10 s, and fluorescence was collected at 60º for 30 s. The primers were checked to be in a range of 90 to 110% efficiency, and the melting temperature (TM) was optimized at 60 °C. Each kinetic time point was analyzed in triplicate. The data were analyzed using the ΔΔCt methodology, using the expression of Alu repeats as a reference gene.

#### Primers used include

KLK6: forward (5’–3’) (AGCAGATGGTGATTTCCCTG, 20 bp) and reverse primer (5’–3’) (ACTTCTCATCCCCAGCACAC, 20 bp); EAR (Expressed Alu Repeats): forward (5’–3’) (GAGGCTGAGGCAGGAGAATCG, 21 bp) and reverse primer (5’–3’) (GTCGCCCAGGCTGGAGTG, 18 bp); ICP0: forward (5’–3’) (GTCGCCTTACGTGAACAAGAC, 21 bp) and reverse primer (5’–3’) (GTCGCCATGTTTCCCGTCTG, 20 bp).

### Indirect immunofluorescence (IFI)

H4 cells were grown in monolayer with 70% confluence on round coverslips placed inside 24-well culture plates. After infection and according to the kinetics, M, 8 and 18 hpi, they were fixed in methanol, blocked in SUPERBLOCK blocking solution EDTA (0.5 M), fish gelatin (1%), BSA (1%), horse serum (1%) and H2Od to 10mL at 4 °C. Incubated with anti-KLK6, anti-ICP8 at 37 °C for 30 min, and then washed with PBS Ca2 + Mg2+ 1X. The secondary antibodies were conjugated to Alexa fluorophores (488 and 594) and incubated at 37 °C for 30 min. They were then incubated with DAPI. The coverslips were mounted with DAKO Fluorescent mounting media (DAKO) and analyzed under a microscope Axio Scope A1 epifluorescence microscope (Carl Zeiss). Images were processed using Fiji software (ImageJ). 8-bit images were analyzed with ICY software (http://icy.bioimageanalysis.org/), using graphical programming to automate cell segmentation, signal/noise threshold calculation, and quantification of average fluorescence intensity per cell.

### Western blot

For the preparation of soluble protein extracts, the cells were washed in ice-cold phosphate-buffered saline (PBS), harvested, and lysed at 4 °C in commercial RIPA buffer 1 × (50 mM Tris Buffer pH 7.5, 150 mM NaCl, 5 mM EDTA, 1% vol/vol NP-40, 0.5% wt/volsodium deoxycholate, and 0.1% wt/vol SDS) (Sigma-Aldrich) supplemented with HALT phosphatase and protease cocktail inhibitor (Thermo Fisher Scientific). All lysates were cleared by centrifugation at 18,000 × g for 20 min at 4 ◦C, and protein concentration was determined with a PierceTM BCA Protein Assay Kit (Thermo Fisher Scientific). Samples with an equal amount of protein were boiled with SDS-PAGE sample buffer 4 × (200 mM Tris Buffer pH 6.8, 8% wt/vol SDS, 40% vol/vol Glycerol, 120 mg Bromophenol blue, and 10% vol/vol β-Mercaptoethanol) for 5 min and then analyzed by SDS-PAGE. The proteins were electroblotted onto Protran^®^ immunoblotting nitrocellulose membranes pore size 0.2 μm (GE Healthcare, Chicago, IL, USA) and then blocked by incubation at room temperature with Bovine serum albumin (BSA) Fraction V 3% for 1 h. Primary antibody was incubated overnight 4 °C and the secondary antibodies for 1 h at room temperature, both antibodies were diluted in PBS 5% (wt/vol) BSA. Chemiluminescence protein detection was performed using SuperSignalTM West Pico PLUS substrate (Thermo Fisher Scientific) or Westar Supernova (Cyanagen, Bologna, Italy) based on the desired level of sensitivity, and the signal was registered using iBrightTM Immunoblot Imaging System (Thermo Fisher Scientific).

### Protein precipitation

The kinetics of infection with HSV-1 in HaCaT cells were aimed at protein extraction, both from the pellet and the supernatant of the cultures, with the same purpose. The supernatant was precipitated with trichloroacetic acid (TCA) and acetone. For this purpose, the supernatant was collected from each point of the kinetics, then centrifuged at 10,000 x g for 30 min in a Micro 220 R Centrifuge (Hettich) to eliminate dead cells and debris, then the supernatant was rescued, and 20% TCA was added in a v/v ratio, to achieve a final concentration of 10% TCA. Subsequently, the sample was vortexed and allowed to precipitate on ice for 40 min. Then, it was centrifuged at 10,000 x g for 15 min at 4 °C in a Mikro 220 R Centrifuge (Hettich). The supernatant was then quickly and carefully aspirated, taking care not to touch the pellet, which should be whitish and spongy. The pellet was washed 3 times with 500 µl of cold acetone (Merck) (-20), centrifuging at 10,000 x g for 5 min at 4 °C each time, to eliminate any remaining TCA from the sample. Finally, the pellet was allowed to air dry, then resuspended in 25 µl of 2 x SDS page buffer and heated to 100 °C for 5 min. They were then separated by electrophoresis in SDS-polyacrylamide gels, then electrotransferred to a nitrocellulose membrane (0.2 μm pore) to then be subjected to immunodetection by the aforementioned antibodies. Chemiluminescence of the enzymatic reaction was visualized using SuperSignal™ West Pico PLUS Chemiluminescent Substrate (Thermo Scientific™) on iBright Imaging Systems (Thermo Scientific TM).

### Polyacrylamide gel electrophoresis

A 10 and 15% polyacrylamide gel was prepared under denaturing conditions. The polyacrylamide separating gel was prepared with Separating Gel Buffer (0.4% SDS; 1.5 M Tris-HCl pH 8.8, 0.04% Smonium Persulfate (PSA 10%) and 0.04% TEMED. The 3.8% polyacrylamide spacer gel was prepared with Spacer Gel Buffer (0.4% SDS; 0.5 M Tris-HCl pH 6.8), 0.01% PSA 10%, 0.04% TEMED. 20 µg of the proteins were taken and 4X Loading Buffer (4X: 4% SDS; 25% glycerol; 0.313 M Tris pH 6.8; 0.7 M β-mercaptoethanol; Bromophenol Blue) was added, then heated at 95 °C for 7 min. The samples were loaded into the gel and migrated using a constant amperage of 0.04 A for 1 h and 30 min (10% gel) and 4 h (15% gel) in 1X Running Buffer (5X Running Buffer: 0.5 M Tris; 0.768 M Glycine; 0.4% SDS). 

### Statistical analysis

For statistical analyses with patient data, IBM SPSS Statistics Version 29 and R (2024, R Foundation for Statistical Computing, Vienna, Austria) was used. Mean differences were compared using Student’s t-test, Mann-Whitney U or chi-squared test depending on the distribution of the variables. Spearman-Rho or Pearson correlation two-tailed analyses were calculated depending on the statistical distribution of the variables. For correlation analyses, controlling for multiple testing was done with Bonferroni method.

Associations of various biomarkers with viral AI were investigated using univariate or multivariate regression analyses with a significance level of 0.05. Moderation effects of the independent variables and effect sizes were analyzed by linear regression and simple slope analyses. In the resulting models, clinical parameters with known association to AD pathology (age, sex, global CDR, APOE epsilon 4 allele frequency) were stepwise included as independent variables and remained in the model when the adjusted R2 increased with an F likelihood of less than 0.05.

For statistical analyses from in vitro assays from cell culture GraphPad Prism 8.0 was used. Fluorescence intensity, relative quantification of gene expression and densitometric analyses for protein levels were analyzed by One-Way ANOVA, followed by Tukey’s test to evaluate pair-wise comparisons. Remaining of KLK6 with and without BafA1 treatment was analyzed by Two-Way ANOVA, followed by Sidak’s test to compare the mean differences between groups that have been split on two independent variables. Significance level was set at *p* < 0.05.

## Results

### Correlation of KLKs with AD biomarkers

**Fig. 1:**
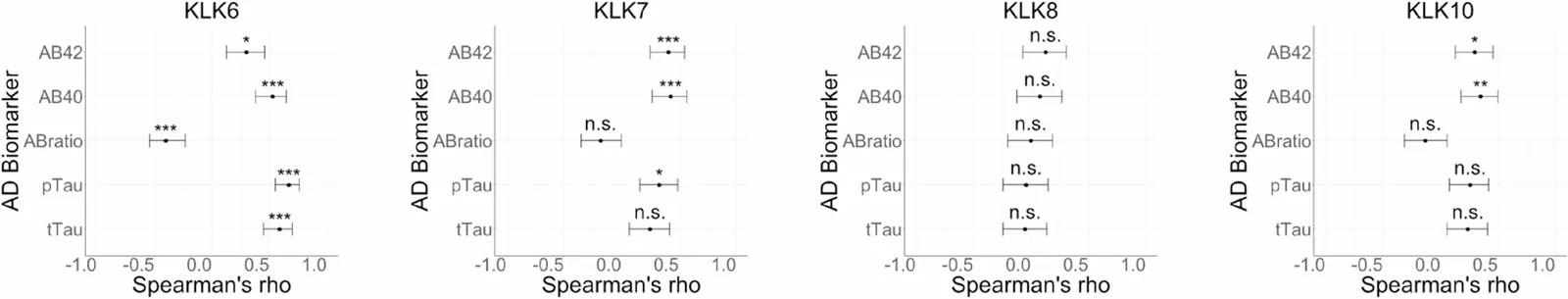
Correlations of CSF-KLKs with CSF AD biomarkers. Correlation coefficients are indicated with 95% confidence interval for 114 patients (102 for KLK8). Significant p-values are indicated with asterisks with a Bonferroni factor of 36 for multiple comparisons (*p≤ 0.05, **p≤ 0.01, ***p≤ 0.001, not significant (n.s.))

In 114 patients with AD (Table 1), the concentrations of KLK6-8 and 10 in the CSF were measured by ELISA and KLK concentrations were correlated with the CSF AD biomarkers. All KLKs except KLK8 are significantly and positively correlated with Aβ42 and Aβ40. Especially KLK6 was significantly and positively correlated with pTau181 and tTau, KLK7 only with pTau181 (Fig. 1). Among AD biomarkers, strongest correlations were found between Aβ40 and Aβ42, Aβ40 and pTau181, as well as between Aβ40 and tTau, and among KLKs between KLK6 and KLK7, KLK6 and KLK10, and KLK7 and KLK8 (Additional file 1: Fig. 1A).

**Table 1:** Patient cohort

Variable	mean ± SD (range), or as absolute numbers
Herpesvirus antibody variables	
Anti-HSV-1-IgG seropositivity	78.9% (*n* = 90 of 114)
Intrathecal anti-HSV-1/2 immune response (HSV-AI) [HSV- / HSV + AI < 1.5 / HSV + AI ≥ 1.5]	24 / 58 / 22 (10 cases not defined)
Anti-HSV-1/2-IgG antibody index in HSV-1-seropositive patients	1.3 ± 0.436 (0.6–2.9; *n* = 80)
Anti-CMV-IgG seropositivity	43.0% (*n* = 49) (1 case not defined)
Intrathecal anti-CMV immune response (CMV-AI) [CMV- / CMV + AI < 1.5 / CMV + AI ≥ 1.5]	64 / 32 / 5 (13 cases not defined)
Anti-CMV-IgG antibody index in CMV-seropositive patients	1.1 ± 0.73 (0.5–3.8; *n* = 37)
CSF AD biomarkers	
Aβ42 [ng/l]	477 ± 140.7 (228–909)
Aβ40 [ng/l] x 10E3	13,246 ± 4330.3 (5060–26423)
Aβ42/ Aβ40 ratio	0.04 ± 0.008 (0.02–0.06)
pTau [ng/l]	92 ± 44.3 (32–231)
tTau [ng/l]	794 ± 473.7 (191–3047)
CSF kallikrein-related peptidases	
KLK6 [ng/ml]	368 ± 128.6 (187–930)
KLK7 [ng/ml]	2.1 ± 0.98 (0.8–6.1)
KLK8 [ng/ml]	0.5 ± 0.18 (0.02–0.12)
KLK10 [ng/ml]	0.52 ± 0.230 (0.06–1.34)
Demographic data	
Age [years]	68.8 ± 8.42 (48–87)
Sex [f/m]	60 / 54 (52.6% female)
MMSE	24 ± 3.7 (13–30)
Global CDR [0/0.5/1/2/3]	0.77 ± 0.340 (0.5-2.0) 0 / 61 / 49 / 2
APOE ε4 alleles [0/1/2]	44 / 48 / 20 (2 cases not defined)
CSF/serum albumin ratio	7.0 ± 3.11 (3.3–26.5)
Total CSF/serum IgG ratio	3.2 ± 1.55 (1.3–13.5)

If HSV-AI or CMV-AI are not defined in HSV + or CMV+, they could not be determined within range (i.e. below 0.5). HSV-: anti-HSV-1-IgG seronegative, HSV + AI < 1.5: anti-HSV-1-IgG seropositive with an anti-HSV-1/2-IgG antibody index below 1.5, HSV + AI ≥ 1.5: seropositive with an anti-HSV-1/2-IgG antibody index equal or higher than 1.5

*AD* Alzheimer’s disease, *Aβ42 or Aβ40* Amyloid 1–42 or amyloid 1–40, *ApoE* Apolipoprotein E, *HSV-AI* CSF to serum anti-HSV-1/2-IgG antibody index, *CDR* Clinical Dementia Rating scale, *CSF* Cerebrospinal fluid, *HSV-1* Herpes simplex virus type 1, *KLK* kallikrein-related peptidase, *MMSE* Mini-Mental state examination, *pTau181* Phospho Tau 181, *tTau* total Tau

### Association of KLKs with AD biomarkers modulated by herpesvirus seropositivity

**Fig. 2:**
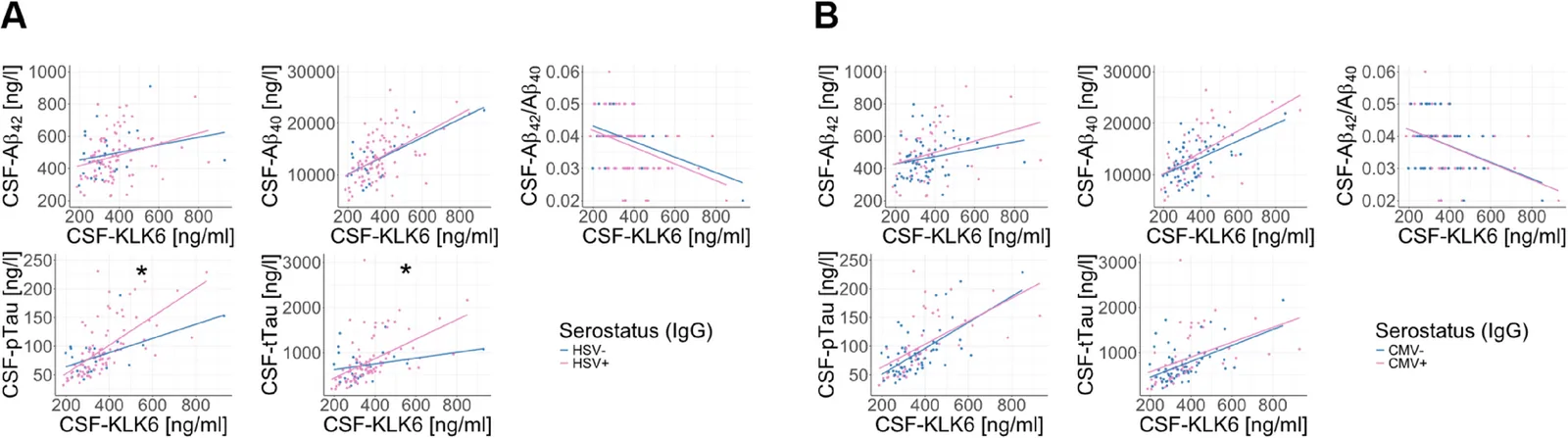
Associations of CSF-KLKs with CSF AD biomarkers stratified by seropositivity of HSV-1, or CMV, respectively. In the linear model with CSF-KLK6 as independent variable and each CSF AD biomarker as dependent, only the associations of CSF-KLK6 with CSF-pTau (adjusted R²=0.381, *p* < 0.001, β_KLK6_ = 0.622, p_KLK6_ = < 0.001) and CSF-tTau (adjusted R²=0.211, *p* < 0.001, β_KLK6_ = 0.467, p_KLK6_<0.001) were significantly modulated by (**A**) HSV-1 seropositivity (pTau: adjusted R²=0.402, *p* < 0.001, β_KLK6_ = 0.358, p_KLK6_=0.009, β_HSV+_= -0.343, p_HSV+_=0.082, β_[HSV+xKLK6]_ = 0.530, p_[HSV+xKLK6]_ = 0.025; tTau: adjusted R²=0.235, *p* < 0.001, β_KLK6_ = 0.172, p_KLK6_=0.261, β_HSV+_=-0.422, pHSV + = 0.059, β_[HSV+xKLK6]_ = 0.600, p_[HSV+xKLK6]_ = 0.025) but not by (**B**) CMV seropositivity (pTau: p_[CMV+xKLK6]_ = 0.615; tTau: p_[CMV+xKLK6]_ = 0.840)

To investigate whether KLKs are associated with a recurrent infection with an alpha- (HSV-1) or betaherpesvirus (CMV), we first determined specific anti-viral-IgG serum antibodies to stratify the cohort according to HSV-1, or to CMV seropositivity, respectively (Table 2, Additional file 1: Table 1). In the next step, linear regression with each AD biomarker as dependent variables and each KLKs as independent variables were performed and controlled for modulating effects of herpesvirus seropositivity. The positive associations of KLK6 with pTau181, or tTau, respectively, were significantly and positively modulated by HSV-1 seropositivity (Fig. 2A) which was not the case for the strong association of Aβ and KLK6, nor of any other KLK (*p* values > 0.5).

**Table 2:** Stratification of patient cohort by intrathecal anti-HSV-1/2 immune response

total *n* = 104^§^	AD HSV- *n* = 24	AD HSV + AI < 1.5 *n* = 58	AD HSV + AI ≥ 1.5 *n* = 22	*p* value
CSF AD biomarkers [mean ± SD (range)]				
Aβ42 [ng/l]	486.2 ± 145.1 (294–909)	470.0 ± 146.26 (228–797)	485.5 ± 136.39 (288–844)	0.941
Aβ40 [ng/l] x 10E3	13 ± 4.0 (7–23)	13 ± 4.6 (5–26)	14 ± 4.7 (7–24)	0.527
Aβ42/ Aβ40 ratio	0.04 ± 0.01 (0.02–0.05)	0.04 ± 0.01 (0.02–0.06)	0.04 ± 0.01 (0.02–0.05)	0.269
pTau181 [ng/l]	82 ± 34 (42–189)	85 ± 38 (32–213)	122 ± 60 (46–231)	< 0.024
tTau [ng/l]	719 ± 354 (270–1573)	702 ± 358 (191–1723)	1115 ± 717 (238–3047)	< 0.039
CSF kallikrein-related peptidases [mean ± SD (range)]				
KLK6 [ng/ml]	347 ± 154 (198–930)	347 ± 92 (187–585)	431 ± 167 (256–850)	0.056
KLK7 [ng/ml]	1.8 ± 0.78 (0.8–4.4)	2.1 ± 0.76 (0.8–4.2)	2.3 ± 1.44 (1.1–6.1))	0.218
KLK8 [ng/ml]	0.05 ± 0.02 (0.02–0.09)	0.05 ± 0.02 (0.02–0.12)	0.05 ± 0.02 (0.02–0.10)	0.954
KLK10 [ng/ml]	0.6 ± 0.30 (0.12–1.34)	0.5 ± 0.24 (0.06–0.97)	0.5 ± 0.2 (0.02–0.10)	0.589
Demographic data [mean ± SD (range), or as absolute numbers]				
Age [years]	67 ± 9 (54–83)	69 ± 9 (48–87)	69 ± 9 (50–83)	0.731
Sex [f/m]	10 / 14	34/24	12/10	0.374*
MMSE	23 ± 5 (13–30)	24 ± 3 (14–30)	24 ± 4 (14–29)	0.774
Global CDR [0/0.5/1/2/3]	0/12/10/2	0/26/28/4	0/7/14/1	0.665*
APOE ε4 allele frequency [0/1/2]	10/10/4	23/23/11	7/11/4	0.941*
CSF/serum albumin ratio	6.6 ± 2.17 (3.3–11.9)	7.1 ± 2.80 (3.5–17.2)	7.4 ± 4.84 (3.3–26.5)	0.972
Total CSF/serum IgG ratio	3.1 ± 1.11 (1.3–6.1)	3.3 ± 1.40 (1.3–7.9)	3.4 ± 2.43 (1.3–13.5)	0.922

After stratification of the patient cohort, differences were tested between all groups using ANOVA or asymptotic Pearson chi square test (asterisks) dependent on the variable‘s metric. In 10 HSV-1-seropositive patients (§) HSV-AI could not be determined within range (i.e below 0.5)

*AD* Alzheimer’s disease, *Aβ42 or Aβ40* Amyloid 1-42 or amyloid 1-40, *ApoE* Apolipoprotein E, *AI-IgG-HSV-1/2* CSF to serum anti-HSV-1/2-IgG antibody index, *CDR* Clinical Dementia Rating scale, *CSF* Cerebrospinal fluid, *HSV-1* Herpes simplex virus type 1, *KLK* Kallikrein-related peptidase, *MMSE* Mini-Mental state examination, *MWU* Mann-Whitney-U test, *pTau181* Phospho Tau 181, *tTau* Total Tau

*Asymptotic Pearson chi square test

CMV seropositivity did not modulate the associations of CSF-KLKs with any AD biomarker, shown for CSF-KLK6 in Fig. 2B. Interestingly, in HSV-1 seropositives, all KLKs were significantly and positively correlated with each other (except of the correlation between CSF-KLK8 with CSF-KLK10) but in the HSV-1 seronegative group only the correlation between CSF-KLK10 and CSF-KLK6 was significant (Additional file 1: Fig. 1B).

### Association of KLK6 with intrathecal anti-herpesvirus immune response

In HSV-1-seropositive (*n* = 80) or CMV-seropositive patients (*n* = 37), anti-herpesvirus-IgG AI (HSV-AI, or CMV-AI, respectively) were measured as an indicator for herpes virus activity in the central nervous system. In a linear regression model, HSV-AI significantly explained variability of CSF-KLK6 but at a very low level (adjusted R²=0.081, *p* = 0.006, β = 0.304) but not CSF-KLK7-8 or 10. As the residuals of this model were not normally distributed, we controlled the model by bootstrapping (10,000 bootstraps, *p* = 0.019, regression coefficient 85.5, confidence interval 16.3-145.7).

**Fig. 3:**
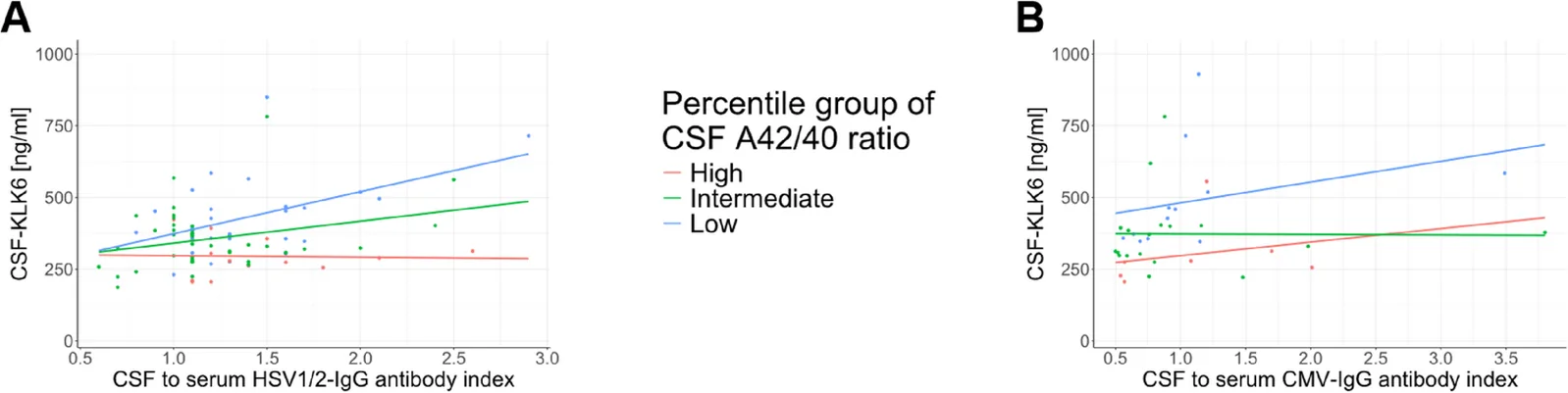
Association of the anti-viral AI of HSV-1/2 or CMV with CSF-KLK6 stratified by tertiles of the CSF-Aβ42/40-ratio. Exploratory visualization of the association between the HSV antibody index and CSF-KLK6 across tertiles of the CSF Aβ42/40 ratio. The association between CSF-KLK6 and HSV-AI in HSV-1-seropositive (**A**) but not between CSF-KLK6 and CMV-AI in CMV-seropositive patients with AD (**B**) was significant. Furthermore, the strength of this association appeared to vary across tertiles of the CSF Aβ42/40 ratio (low, intermediate, and high), although the interaction term did not reach statistical significance. (displayed separately for the low, intermediate, and high tertiles). Low: adjusted R²=0.137, *p* = 0.025, β = 0.408; intermediate: adjusted R²=0.121, *p* = 0.035, β = 0.349; high: adjusted R²=-0.068, *p* = 0.638, β = 0.144

In the next step, we investigated whether the association of CSF-KLK6 with HSV-AI was further significantly modulated by the CSF-Aβ42/40-ratio (*p* < 0.001, Fig. 3A). This was investigated as in this cohort the association of HSV-AI with CSF-pTau181 was significantly modulated by the CSF-Aβ42/40-ratio [20], and as CSF-pTau181 correlates strongly with CSF-KLK6. Therefore, we first included the CSF-Aβ42/40-ratio as an additional independent variable which increased the explained variability of the model (adjusted R²=0.270, *p* < 0.001, β_HSV−AI_ = 0.306, p_HSV−AI_=0.002; β_abeta−ratio_=-0.442, p_abeta−ratio_<0.001). Addition of the CSF Aβ42/40 ratio substantially improved the model fit (adjusted R² = 0.270), with both variables remaining independently associated with CSF-KLK6. Inclusion of the interaction term resulted in only a modest further increase in explained variance (adjusted R² = 0.291, *p* < 0.001), while the interaction itself showed only a statistical trend (β_HSV−AI_ = 1.216, p_HSV−AI_= 0.020, β_abeta−ratio_= -0.188, p_abeta−ratio_=0.603, β_HSV−AIxabeta−ratio=_-1.122, p_HSV−AIxabeta−ratio_=0.077). Additional regression diagnostics supported the robustness of the multivariable regression model (Additional file 1: Table 2). After centering the predictors, no relevant multicollinearity was observed (all variance inflation factors < 1.1). Although four observations showed moderately increased Cook’s distances and two observations had externally studentized residuals exceeding ± 3, no single observation appeared to disproportionately influence the model estimates.

CMV-AI was not associated with any CSF-KLKs, displayed for KLK6 (Fig. 3B).

CSF-KLK6 levels were also compared between seronegative and seropositive patients stratified to normal or increased intrathecal anti-HSV-1/2 immune response (Additional file 1: Fig. 2).

### Moderator analyses reveal direct and indirect effects of HSV + AI on CSF-tau biomarkers

To examine whether HSV-1-associated neurotoxicity is mediated by KLK6 or merely reflects auto-correlated increases of KLK6 and tau, we conducted a moderator analysis. In this analysis CSF-pTau181, or CSF-tTau, respectively, are dependent variables, HSV AI is the independent variable and CSF-KLK6 is the moderator variable. For both models significant direct and indirect effects via CSF-KLK6 on pTau, or tTau, respectively, were determinated for HSV AI (Additional file 1: Fig. 3A).

### Prognostic CSF-KLK6 on longitudinal AD biomarker alterations

In 18 patients (compare Table 3), AD biomarkers were measured in a longitudinal CSF assessment (two lumbar punctures with an average interval of 0.98 years (range 0.3-2.0, SD = ± 0.58). To investigate effects of CSF-KLKs at first baseline CSF assessment on alterations in AD biomarkers per year (delta, Δ), Spearman-Rho correlation analyses were performed. Accounting for the subgroup sizes (HSV- *n* = 1, HSV + AI < 1.5 *n* = 13, HSV + AI ≥ 1.5 group *n* = 4), only the HSV-seropositive patients were investigated (*n* = 17). CSF-KLK6 at baseline was inversely correlated with ΔCSF-Aβ42 (*n* = 17), ΔCSF-Aβ40 (*n* = 15, not available in two cases) and positively with ΔCSF-tTau (*n* = 17) but not with ΔCSF-pTau (*n* = 17) (Additional file 1: Fig. 3B). Of all other CSF-KLKs, only CSF-KLK8 (*n* = 16, one value below detection limit) was positively and significantly correlated with ΔCSF-Aβ42 (Rho = 0.498, *p* = 0.050) and inversely with ΔCSF-tTau (Rho=-0.515, *p* = 0.041) (Additional file 1: Fig. 3C).

**Table 3:** Patients’ characteristics of the longitudinal subgroup

Variable	Mean change per year ± SD (range)
number	18
ΔCSF-Aβ42 [ng/l]	-54 ± 195.9 (-461 – +323)
ΔCSF-Aβ40 [ng/l]	-1403 ± 4230 (-10722–6148)
ΔCSF-Aβ42/40 ratio	0.1 ± 0.013 (-0.03–0.03)
ΔCSF-tTau [ng/l]	159 ± 655.7 (-543–2654)
ΔCSF-pTau181 [ng/l]	-1.4 ± 9.69 (-23.2–15.6)
Years between lumbar punctures	0.9 ± 0.58 (0.3–2.0)
Anti-HSV-1-IgG seropositivity	94.4% (*n* = 17)
HSV-AI (negative / normal / elevated / no data) at baseline	1.3 ± 0.48 (0.6–2.5) (*n* = 16) 1 / 6 / 4 (≥ 1.5) / 1
ΔCSF/serum albumin ratio x 10³	-0.25 ± 1.57 (-4.5–2.36)
ΔCSF/serum IgG ratio x 10³	-0.61 ± 3.51 (-8.8–4.24)

*Aβ42 or Aβ40* Amyloid 1–42 or amyloid 1–40, *HSV-AI* CSF to serum anti-HSV-1/2-IgG antibody index, *CSF* Cerebrospinal fluid, *HSV-1* Herpes simplex virus type 1, *pTau181* Phospho Tau 181, *tTau* total Tau

### In vitro KLK protein expression in various human cell models after HSV-1 infection

**Fig. 4:**
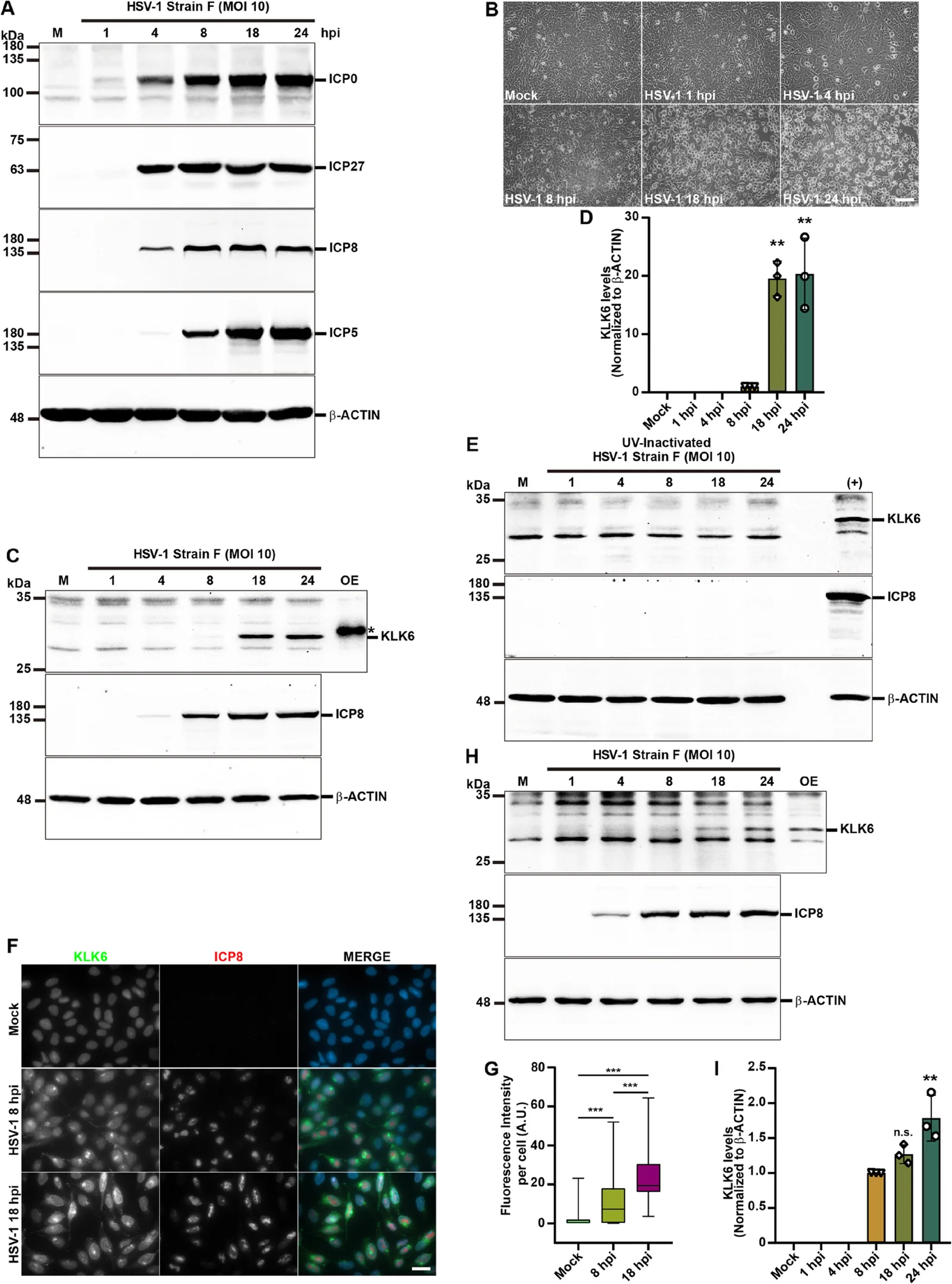
Expression of HSV-1 proteins and KLK6 in neuronal-like cell lines during productive infection. Western blot analyses show (**A**) HSV-1 proteins (ICP0, ICP27, ICP8 and ICP5) and housekeeping protein (β-ACTIN), in uninfected (Mock or M) or HSV-1-infected H4 cells at a multiplicity of infection (MOI = 10). Analyses were performed at 1, 4, 8, 18 and 24 h post infection (hpi). **B** Cellular morphology and cytopathic effects of uninfected (Mock) and HSV-1-infected H4 cells were recorded by bright- field microscopy, magnification is 80-fold. Scale bar = 50 μm. **C**-**D** KLK6 protein in uninfected or HSV-1-infected H4 cells, and H4 cells transfected with pCMV-SPORT6-KLK6 as positive control (OE). **E** Infection kinetics in H4 cells with UV-inactivated HSV-1, (+) H4 cells infected at 18 hpi with non-UV-irradiated HSV-1. **F**-**G** Indirect immunofluorescence showing KLK6 (green Alexa Fluor 488), viral protein (ICP8) (red Alexa Fluor 594), and DAPI staining of nuclei, in uninfected (Mock), or HSV-1-infected H4 cells at 8 and 18 hpi, magnification is 630-fold. Quantitative analysis of the mean of fluorescence intensity of KLK6 (Mock 104 cells; HSV-1 8 hpi 124 cells; HSV-1 18 hpi 63 cells). **H**-**I** KLK6 protein in uninfected (Mock or M) or HSV-1-infected SH-SY5Y cells at a multiplicity of infection (MOI = 10) and positive control KLK6 overexpression. **D** and **I** Results were expressed as the ratio of KLK6 to β-ACTIN protein levels. Western blots are representative of three independent experiments

Our clinical data in AD suggests that intrathecal anti-HSV-1 immune response may be linked to KLK6 concentration in the CSF. Therefore, we investigated if there is a functional association in vitro. In human neuroglioma cells (H4) immediate early viral proteins ICP27, ICP0, early viral protein ICP8 and late viral protein ICP5 could be detected by Western blot from 4 to 8 until 24 h post-infection (hpi), aligned with cytopathic effects (Fig. 4A-B). β-Actin was used for protein normalization. As positive control for KLK6 protein detection, we overexpressed KLK6 in H4 cells (DU57546, pCMV-SPORT6-KLK6). Induction of KLK6 protein expression was confirmed in H4 cells from 8 hpi onwards with HSV-1 (Strain F) with a MOI of 10 (Fig. 4C-D) and KLK6 was not detected in H4 cells infected with UV-inactivated HSV-1 (Fig. 4E).

Furthermore, through immunofluorescence imaging, we analyzed the distribution of KLK6 in H4 cells. KLK6 exhibited a pan-cellular distribution, with a predominantly perinuclear punctiform pattern during productive HSV-1 infection. Furthermore, the staining intensity of KLK6 was induced during HSV-1 infection (Fig. 4F) with increased fluorescence intensity in HSV-1 infected cells at 18 hpi (Fig. 4G), with no apparent overlap between the channels analyzed. Also, in human neuroblastoma cells (SH-SY5Y) KLK6 protein was induced in late stages of HSV-1 infection, as ICP8 increases (Fig. 4H-I).

**Fig. 5:**
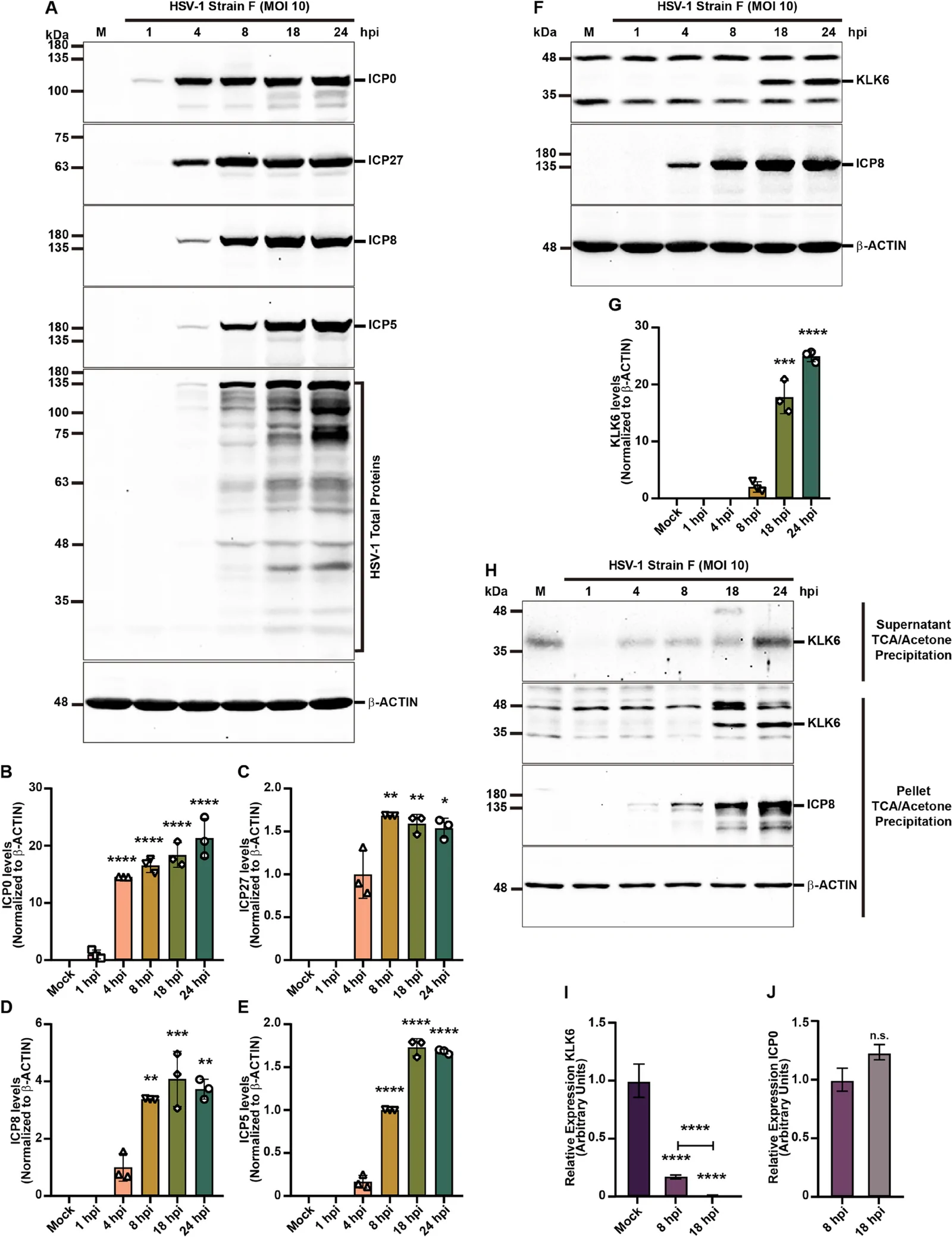
Expression of HSV-1 proteins and KLK6 in human keratinocytes cell line during productive infection. Western blot analyses show (**A**) HSV-1 proteins (ICP0, ICP27, ICP8, ICP5, and HSV-1 total proteins) and housekeeping protein (β-ACTIN) in uninfected (Mock or M) or HSV-1-infected HaCaT cells and their respective densitrometric analysis (**B-E**). **F** KLK6, HSV-1 protein (ICP8), and housekeeping protein (β-ACTIN). **G** Densitometric analysis of KLK6. **H** KLK6 protein in the extracellular medium during HSV-1 infection precipitated with TCA/Acetone and KLK6, ICP8 and β-ACTIN proteins precipitated with TCA/Acetone in pellet of uninfected (Mock o M) and HSV-1-infected HaCaT cells. All kinetics were performed at a multiplicity of infection (MOI = 10). **I** KLK6 mRNA levels and (**J**) viral ICP0 mRNA were analyzed by RT-qPCR in uninfected (Mock or M) or HSV-1-infected HaCaT cells at multiplicity of infection (MOI = 10). Infection was performed at 8 and 18 h post infection (hpi). Relative mRNA expression of KLK6 and ICP0 were normalized to Expressed Alu repeats (EAR) sequences. Data were analyzed using the ΔΔCt methodology, using EAR expression as reference for RTqPCR [67]. **A-G** Analyses were performed at 1, 4, 8, 18 and 24 h post infection (hpi). Results were expressed as the ratio of KLK6 or a specific viral protein to β-ACTIN protein levels. All results are representative of three independent experiments

Human keratinocytes (HaCaT) Western blot analyses show immediate early viral proteins ICP27, ICP0, early ICP8 and late ICP5 normalized to β-Actin after infection with HSV-1 (Fig. 5A), demonstrated in the respective densitometric analyses (Fig. 5B-E). Interestingly, the induction of KLK6 protein expression begins at 8 hpi and increases sharply at 18 hpi, being undetectable in uninfected cells (Fig. 5F-G).

In addition, we also investigated the intracellular levels of five other KLKs (Additional file 1: Fig. 4A-G). We found two different phenotypes, KLK1 and KLK12 also were induced during HSV-1 infection from 8 hpi and 4 hpi, respectively (Additional file 1: Fig. 4A-C), and KLK5, KLK7 and KLK10 are constitutively expressed proteins and increase their levels during HSV-1 infection (Suppl. Figure 4D-G). This upregulation in KLKs correlates with findings in VZV [33], in which the ubiquitin ligase MDM2 was coexpressed with KLK6 and promoted keratin 10 (KRT10) degradation through binding and subsequent targeting to the ubiquitin-proteasome pathway. We also found a significant reduction in the protein levels of KRT10 during HSV-1 infection. This decrease was already significant at 24 hpi (Additional file 1: Fig. 4H-I). Also, a decrease of another target of MDM2, the tumor suppressor protein p53, was found at 18 and 24 hpi (Additional file 1: Fig. 4H-J).

#### The temporal mismatch between KLK6 mRNA and protein levels during HSV-1 infection in HaCaT cells

As HSV-1 mainly infects keratinocytes and reenters them upon reactivation from trigeminal ganglia, keratinocytes were chosen to model the link between acute HSV-1 infection and KLK6.

Interestingly, although the KLK6 protein levels increased in all used cell models, whether measured in cell pellets or KLK6 secreted in the supernatants (shown in HaCaT cells, Fig. 5H), post HSV-1 infection, a downregulation of KLK6 mRNA was observed at 8 hpi and was no longer detected at 18 hpi (Fig. 5I) whereas viral ICP0 mRNA expression was not affected (Fig. 5J). However, a high KLK6 mRNA expression was only robustly detected in the HaCaT cell line and not in the neuron-like SH-SY5Y or H4 cell line. In the case of SH-SY5Y, we also differentiated the cell line to a neuron-like type [39] but did not detect KLK6 protein or mRNA expression at 24 hpi. For the stronger relationship between HSV-1 and KLK6 protein expression, HaCaT cells were selected to investigate KLK6 turnover.

**Fig. 6:**
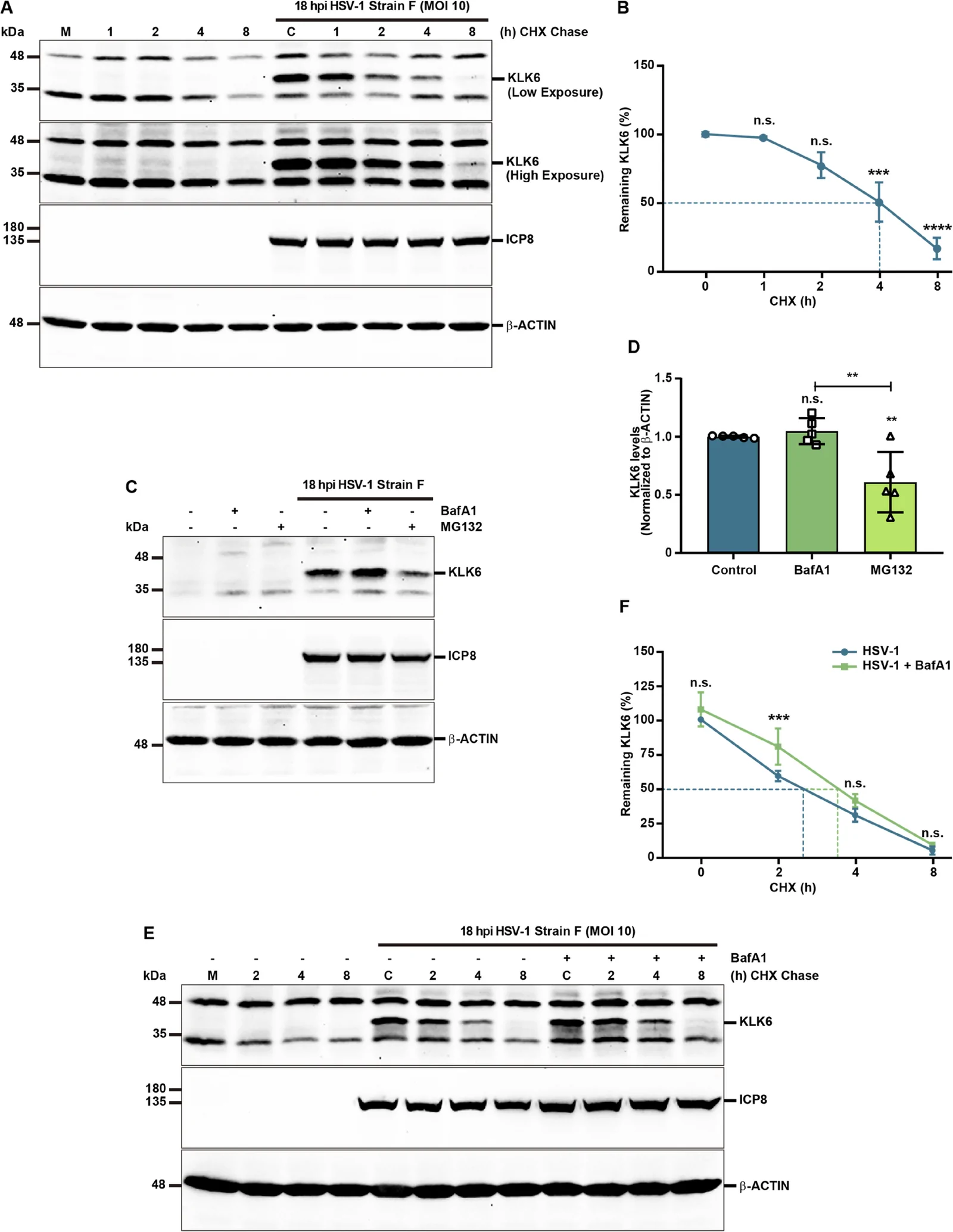
Impact of HSV-1 infection on stability of intracellular levels of KLK6 in HaCaT cells. **A** The effect of HSV-1 infection at 18 hpi on KLK6 protein stability was examined by CHX chase assay in 2-hour interval and chase till 8 h. protein extracts were prepared at the indicated time points after CHX treatment (100 µg/ml) for Western blots analyses. **B** Relative changes in the half-life of KLK6 were quantified and expressed as % remaining. **C** Uninfected and HSV-1-infected HaCaT cells were treated at 10 hpi with BafA1 (160 µM) or MG132 (10 µM) or left untreated for 8 h. **D** Densitrometric analysis of KLK6. **E** Uninfected and HSV-1-infected HaCaT cells were left untreated or treated with CHX alone or with a combination of CHX and BafA1 (CHX + BafA1) and processed as in **A**. **F** Relative changes in the half-life of KLK6 were quantified in HSV-1-infected HaCaT cells with and without BafA1 and expressed as % remaining. All results are representative of three independent experiments

To address potential effects of HSV-1 on KLK6 half-life, we assessed protein stability using CHX chase at 2-hours interval and chase till 8 h (~ 4 h; Fig. 6A–B) and examined degradation via proteasome and lysosomal pathways. As KLK6 mRNA expression has already significantly decreased at 8 hpi (Fig. 5I), we assume that if mRNA is downregulated after 8 hpi, there should be no template for translation at 24 hpi. However, actin-normalized KLK6 protein levels peaked at 24 hpi (Fig. 5G).

Inhibition of lysosomal V-ATPase activity with bafilomycin (BafA1) 18 hpi led to a slight increase in KLK6 protein (Fig. 6C-D). Proteasome inhibition with MG132 significantly reduced KLK6 protein levels, probably due to activation of a compensatory cellular mechanism that degrades KLK6 under MG132 treatment (Fig. 6D). BafA1 prolongs KLK6 half-life significantly (Fig. 6E-F) as consequence of its stabilization during the first hours of CHX-chase (Fig. 6F) in HSV-1 infected keratinocytes, which supports the idea that although KLK6 mRNA expression being early downregulated during viral infection, the protein is stabilized and subsequently degraded preferentially by lysosomal pathway.

#### HSV-1 replicative cycle is dependent on KLK6 expression in HaCaT cells

**Fig. 7:**
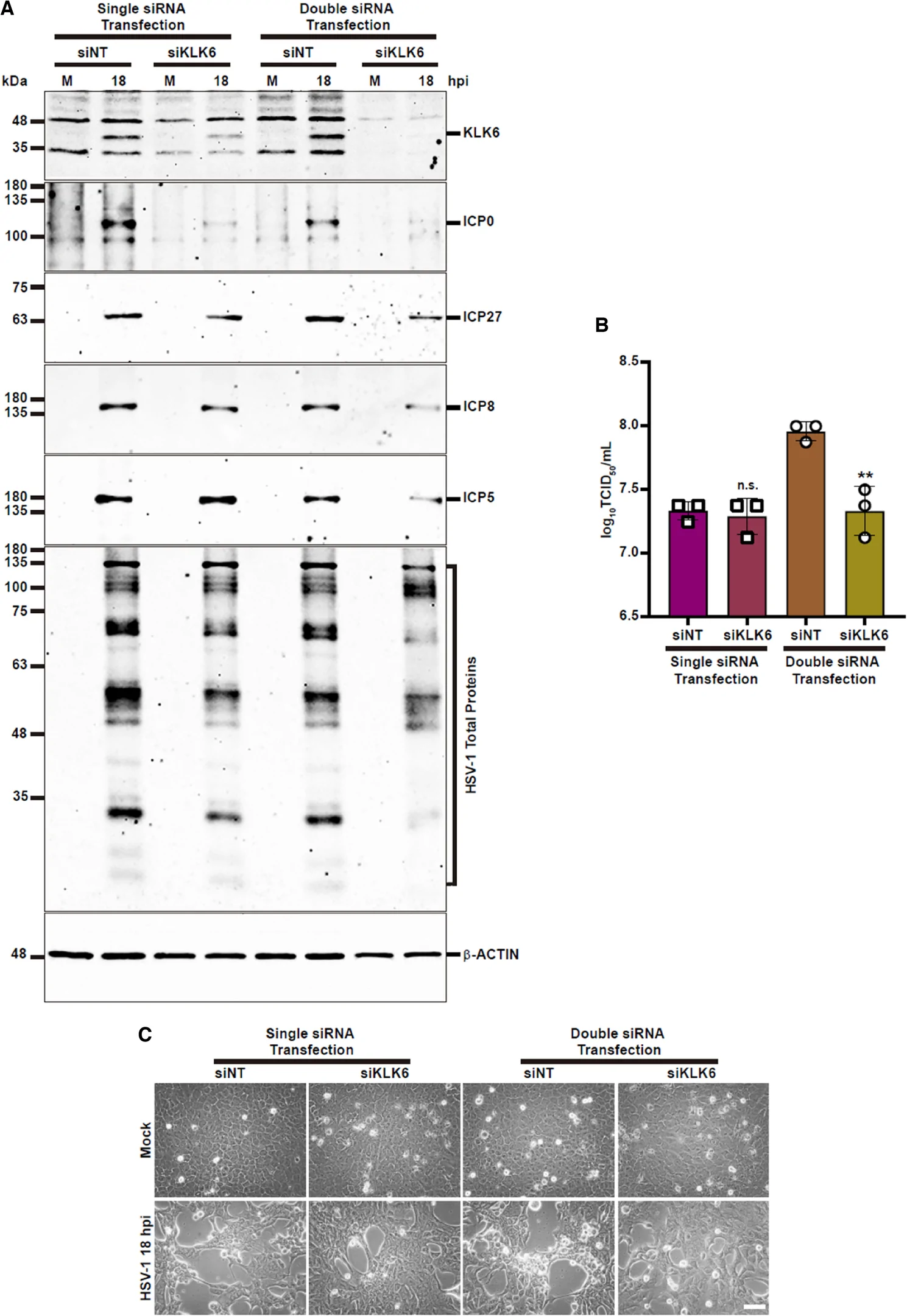
Effect of KLK6 silencing on the HSV-1 replication cycle. Western blot analyses show protein levels of (**A**) KLK6, viral proteins ICP0, ICP27, ICP8, ICP5, HSV-1 total proteins and housekeeping protein (β-Actin) in uninfected (Mock or M) or HSV-1-infected HaCaT cells for 18 hpi at a multiplicity of infection (MOI = 10) either transfected with siNT or transfected with KLK6 siRNA (siKLK6) with 1 and 2 rounds of transfection 100nM each, every 24 h. **B** Progeny HSV-1 yield was assessed by titration in Vero cells through TCID50 at 96 hpi from supernatants derived of HSV-1-infected HaCaT cells shown in panal **A**. **C** Cellular morphology and cytopathic effects of uninfected (Mock) and HSV-1-infected HaCaT cells were recorded by bright- field microscopy, magnification is 160-fold. Scale bar = 20 μm. Cell observation at 18 hpi to phenotypically identify the effect of KLK6 silencing in this type of cells, during HSV-1 infection analyzed in A. All results are representatives of three independent experiments

To clarify whether KLK6 is a bystander product of HSV-1 infection or directly involved in its replication cycle, we used RNA interference for suppression of KLK6 gene expression and evaluated HSV-1 proteins and titers. When silencing KLK6 (siKLK6) in HaCaT cells by two-rounds of transfection every 24 h, a remarkable decrease in viral protein ICP0, ICP27, ICP5 and as a trend in ICP8 can be observed following HSV-1 infection together with an effective depletion of KLK6 protein levels, compared to silencing using a Non-Targeting siRNA (siNT) (Fig. 7A). Knockdown of KLK6 also decreased HSV-1 titer by TCID_50_ assay after double siRNA KLK6 transfection (Fig. 7B). HSV-1 infection not only led to a stabilization of KLK6, but KLK6 also appears to contribute to HSV-1 replication. Additionally, we recorded cell integrity by bright-field microscopy. Remarkably, KLK6 silencing partially prevented the typical cytopathic effect of HSV-1 in both single and double siRNA KLK6 transfection, characterized by the production of lysis foci and cell detachment, in agreement with decline in viral proteins and infectious progeny titer (Fig. 7C).

## Discussion

In this study, we present the first evidence that induced KLK6 expression occurs during HSV-1 infection and that the intracellular stabilization of KLK6 protein is linked to HSV-1 replication. In AD CSF, KLK6 levels were linked to intrathecal anti-HSV immune response (indicated by the anti-HSV-1/2-IgG antibody index (HSV-AI)) and to AD biomarkers both cross-sectionally and longitudinally in a small HSV-1–seropositive exploratory subgroup. This supports the hypothesis that KLK6 can serve as a biomarker for intrathecal anti-HSV immune response in patients with AD. The present findings, together with our previous observations and experimental data, further support the hypothesis that HSV-1-related immune responses may contribute to AD pathophysiology but do not establish causality… KLK6 may be a potential regulator of amyloid homeostasis and HSV-1-associated neurotoxicity. In comparison to other KLKs (KLK7-8 and 10) and betaherpesviruses like CMV, elevated CSF-KLK6 levels are rather specific for the alphaherpesvirus HSV-1.

In the CSF of patients with early AD compared to cognitive healthy controls, we have shown that only KLK6 - unlike KLK8 and KLK10 - was significantly increased and positively correlated with tTau and pTau181 [3]. The positive association of tTau or pTau181 with KLK6 was confirmed in the present study and is not observed for other investigated KLKs (only showed a trend for KLK7). In the CSF, we can also confirm a positive correlation of KLK6 with Aβ42 and Aβ40 initially shown by Patra et al. [29]. KLK6 correlates more strongly with Aβ40 which is rather linked to amyloid turnover than the more fibril-forming amyloid species Aβ42 [40]. We show that only CSF-KLK6 of all investigated KLKs is significantly linked to cerebral Aβ plaque load indicated by its inverse correlation with the CSF-Aβ42/Aβ40 and its positive correlation with elevated CSF-pTau181 levels (which was found to correlate strongly with cerebral amyloid pathology) [40]. The positive correlation with CSF-tTau might indicate that CSF-KLK6 is derived from neuronal death. But as none of the other brain derived and often in various cell lines co-expressed KLKs [33, 41] show such correlation, this explanation is unlikely.

KLK6 may influence soluble amyloid homeostasis via amyloid precursor protein (APP) cleavage [42–44], but its impact on Aβ turnover or other species derived from amyloidogenic processing remains unclear. In the exploratory small longitudinal subgroup, high CSF-KLK6 levels significantly predicted a decrease in the Aβ42 and Aβ40 levels over one year on average, suggesting a possible non-amyloidogenic function of KLK6.

For the first time, CSF-KLK7 and KLK10 were found to correlate positively with soluble Aβ, suggesting a potential class effect of KLKs in amyloid homeostasis despite differing substrate specificities [45].

Focusing on KLK6, this protease is expressed throughout the human brain, with strong staining in hippocampus, frontal lobe, and subcortical nuclei. It can be found in excitatory neurons, including pyramidal neurons, but is predominantly enriched in oligodendrocytes [46]. However, under conditions of inflammation or neuronal injury, KLK6 protein is also highly expressed in astrocytes and microglia [47, 48]. Both cell types are activated in early stages of AD [49, 50] and become functionally impaired in the course of the disease. In the CSF, KLK6 trended to increase with disease progression over a follow-up period of one year but not consistently with longer follow-ups [29]. The transient increase in KLK6 also fits to a rather transient increase in astrocytosis and microglial activation at early AD stages [49, 50]. A raised CSF-KLK6 level may go along with a negative affection of these cell systems linked to progressing Aβ and tau pathology.

Based on the observations that infection of alphaherpesviruses like HSV-1 and VZV can induce transient mRNA expression of various KLKs in different cell models [31, 33], we suspected that CSF-KLK6 has the potential to function as surrogate biomarker for intrathecal immune response. HSV-1 establishes latency primarily in peripheral sensory ganglia. Consistent with the work of Du and Roizman [51] classical HSV-1 latency has not been convincingly demonstrated in CNS neurons, which appear to lack key molecular mechanisms required for the establishment of latency. Consequently, the intrathecal anti-HSV antibody response should not be interpreted as evidence of latent HSV-1 infection or ongoing viral replication within the brain, but rather as a marker of previous or recurrent CNS antigen exposure. But HSV-1 can lytically infect various CNS cell types, including neurons, microglia, and oligodendrocytes [52], potentially contributing to neurodegeneration.

In this cohort, we previously reported a positive association of intrathecal anti-HSV-1/2 immune response with CSF-tTau and CSF-pTau181, strongest in HSV + AI ≥ 1.5 patients and modulated by soluble amyloid [20]. Here, we show that CSF-KLK6 is likewise highest in HSV + AI ≥ 1.5 compared to HSV − and HSV + AI < 1.5. But the HSV antibody index alone explained only a modest proportion of the variance in CSF-KLK6 levels (adjusted R² = 0.081), indicating that the intrathecal anti-HSV immune response is likely one of several biological factors contributing to KLK6 regulation.

In HSV-1–seropositive AD patients, HSV-AI significantly predicted CSF-KLK6. Although the CSF Aβ42/40 ratio as a modulator of this association showed only a statistical trend, its direction was consistent with the hypothesis that the relationship between HSV-associated immune activation and KLK6 may depend on the underlying amyloid pathology, as seen for CSF-pTau. This exploratory finding requires confirmation in independent cohorts. Thus, KLK6 appears closely linked to tau pathology.

Additionally, the previous observed positive association of CSF-KLK6 with CSF-pTau181 and CSF-tTau was significant only in HSV-1-seropositive but not HSV-1 seronegative patients. To further explore whether CSF-KLK6 is merely a consequence of HSV-1-associated neuronal injury and passive release together with tau proteins, we performed mediation analyses. These analyses suggested that the anti-HSV-1/2-IgG antibody index was associated with both direct and indirect effect via CSF-KLK6 on CSF-pTau, and CSF-tTau. Cross-sectionally, CSF-KLK6 appeared to be partly independent of neuronal injury and associated with tau pathology. Longitudinally, higher baseline CSF-KLK6, interpreted with caution because of the small subgroup size, predicted increased CSF-tTau after one year in HSV-1-seropositive patients but not CSF-pTau181. Thus, CSF-KLK6 may reflect processes associated with HSV-1-related injury and tau pathology in HSV-1–seropositive patients. However, the lack of longitudinal data in HSV-1-seronegative individuals precludes conclusions regarding HSV-1-independent effects on AD biomarkers. Although the mediation analyses are consistent with the proposed biological model, they are based on observational data and therefore cannot establish the directionality or causality of the relationships between the intrathecal anti-HSV immune response, KLK6, and tau pathology.

Recent evidence indicating that phosphorylated tau (pTau) can interact with intracellular HSV-1 capsids and restrict viral replication raises the possibility that increased pTau levels may partly reflect an augmented intrinsic antiviral response [52], potentially mirrored by increased CSF-KLK6 levels.

Elevated HSV-AI levels can also be detected in normal controls (NC) without AD where no elevation of CSF tau biomarkers is observed [20], which means that under physiological conditions, lytic infection of bystander cells of neurons following acute HSV-1 reactivation in the brain may be prevented by host defense mechanisms. This could be different in AD, i.e. for a decreased virostatic soluble amyloid concentration in the brain. If confirmed in independent cohorts, CSF-KLK6 might help identify patients for future studies evaluating anti-HSV treatment strategies.

Based on clinical data, we investigated whether HSV-1 infection leads to an increased KLK6 protein expression in three different human cell models: neuron-like neuroblastoma cells (SH-SY5Y), neuroglioma cells (H4) and keratinocyte cells (HaCaT). For investigating the association of KLK6 and HSV-1 infection in vitro, we then selected the keratinocyte cell line HaCaT for the strong KLK6 mRNA expression and because HSV-1 initiates productive infection in skin and mucosal epithelium, especially keratinocyte-lineage cells, before accessing sensory neurites and establishing latency in ganglionic neurons. This makes keratinocytes the biologically relevant first compartment for studying entry, early replication, and epithelial innate responses [68, 69, 70]. None of the cell models establishes HSV1 latency.

KLK6 mRNA expression following HSV-1 infection was transient and significantly decreased after 8 hpi with HSV-1. The strong protein signal with only transient KLK6 mRNA expression was counter-intuitive but had been reported also in a study with the other humanopathogenic alphaherpesvirus VZV [33]. In vitro assays have demonstrated that KLK6 has a short half-life, with its auto-catalytic activation starting at 10 min and complete cleavage to the inactive form occurring at 240 min [53] demonstrating a short half-life.

However, we detected increased KLK6 protein levels in all cell models investigated after HSV-1 infection, even though our data indicates that KLK6 has an intracellular half-life of approximately 4 h under the same conditions. KLK6 protein was not detected in uninfected cells.

It was also observed that at 18 hpi and treated for 8 h with CHX the KLK6 protein was no longer visible, which indicates that at this time, the total protein remnant (post-inhibition of protein synthesis) was degraded, suggesting rapid turnover. Still, we could observe a continuing increase in KLK6 protein expression until more than 24 hpi. To explore whether KLK6 accumulation during HSV-1 infection is due to reduced degradation, we investigated the two main proteolytic pathways: the autophagy-lysosomal pathway (ALP) and the ubiquitin-proteasome system (UPS), responsible for 10–20% and 80–90% of protein degradation, respectively [54]. KLK6 had a slight accumulation in a steady-state, along with a significant increase in its half-life following the vacuolar H+-ATPase inhibitor bafilomycin (BafA1) treatment, but not following the proteasome inhibitor MG132, being in line with a lysosomal degradation of KLK6. MG132 can induce compensatory autophagy [55], as a possible reason why MG132-treatment leads to even a decreased KLK6 protein expression when compared to the untreated condition. During HSV-1-infection, the virus inhibits Beclin-1 by the viral ICP34.5, which is a key regulator of autophagy in the nucleation step [56]. This inhibition likely contributes to KLK6 accumulation during infection [57], which may also impact on APP processing [58]. Inhibition of lysosomal pathways helps HSV-1 to establish an infection as xenophagy serves as a powerful host defense against HSV-1 replication [59]. Although HaCaT keratinocytes are a relevant model for HSV-1 infection, these findings require confirmation in neural cell models.

Recently, it has been shown in the case of VZV infection in a keratinocyte cell model that KLK6 and the ubiquitin ligase MDM2 are simultaneously upregulated during infection [33]. MDM2 binds to keratin 10 (KRT10) and p53, targeting these proteins for degradation via UPS which is critical in restraining the activity of these proteins. Preventing KRT10 degradation reduced VZV propagation in culture cells and prevented epidermal disruption in skin explants [33]. Since we also observed a decrease in KRT10 and p53 protein levels after HSV-1 infection of keratinocytes accompanied by an increase in KLK6, our findings suggest that some components of the MDM2/KRT10 pathway may also be involved during HSV-1 infection. However, the role of p53 during HSV-1 infection appears to be complex and may differ depending on the experimental model and stage of infection. Although Maruzuru et al. reported a more complex [60], context-dependent role of p53 during HSV-1 infection, our data demonstrate an overall reduction of p53 protein abundance at 18 and 24 hpi under our experimental conditions. Considering the observed decline in p53 protein levels during viral replication, altered cell cycle regulation may have contributed to the observed phenotype. According to our data, UPS seems to be less meaningful for the degradation of KLK6 than the lysosomal pathway.

The fact that a consistent but only transient KLK6 mRNA expression can be found in HSV and VZV infection, leading to an increased KLK6 protein abundance, makes the result of this study more plausible. In contrast to MDM2, the critical role of KLK6 during the alphaherpesviruses infection has yet to be uncovered in various cell systems. But we already can show that transient silencing of KLK6 protein expression using siRNA in HaCaT cells, reaching a complete loss of KLK6 protein detection, reduces viral protein levels of ICP0 and ICP8, both crucial for HSV-1 replication, in correlation with a decreased HSV-1 titer. Although silencing did not prevent HSV-1 infection totally, it negatively impacted viral replication. This means that KLK6 seems not to be only a bystander protein but appears to contribute in the HSV-1 progeny. In addition, silencing KLK6 resulted in a reduction of cytopathic effects in HSV-1-infected cells. Considering our finding of declining p53 levels during viral replication, this phenotype could be partly based on general cell arrest. Taken together, the clinical associations and in vitro findings support a biologically plausible link between HSV-1 infection and KLK6. However, the clinical findings are observational, whereas the in vitro data provide functional evidence under controlled conditions and should not be directly extrapolated to patients.

In CSF, only KLK6 correlated significantly with intrathecal anti-HSV-1/2 immune response. KLK7 correlated with KLK6 and AD biomarkers, but showed only a trend with HSV-1 activity, reflecting functional differences despite structural similarity [27]. KLK6 exhibits trypsin-like specificity and KLK7 displays a unique chymotrypsin-like specificity. Still, it might be a lack of power that for KLK7 correlations with HSV-1 activity were not significant.

CSF-KLK8 showed no consistent correlations with AD biomarkers or HSV-AI, and the previously reported negative association with CSF amyloid could not be replicated [3]. Considering that KLK8 in the hippocampus has been negatively associated with Aβ clearance in a mouse model [61], the lack of an association of CSF-KLK8 with CSF Aβ biomarkers suggests no consistent measurable impact of KLK8 on AD. Further, CSF-KLK8 levels did not differ dependent on sex, as increased cerebral KLK8 levels in females recently have been suggested [62]. This is astonishing because CSF-KLK8 has been implicated as a possible biomarker in AD.

No CSF-KLK correlated with intrathecal CMV activity, suggesting KLK6 is more specific for HSV-1 than KLK7-8 and 10. In addition to HSV-1, increased KLK protein expression was also found as a response to the other human pathogenic alpha-herpesvirus VZV [33]. The results of our work and that of others thus point to a specific KLK expression in alphaherpesviruses in general. Compared to most other herpesviruses, this viral subfamily has the ability to replicate relatively quickly, exhibit lytic properties and show a tissue tropism for epithelial mucosa and sensitive neurons.

Our data suggests that KLK6 is partly required for the HSV-1 replication, as knock-down reduces viral activity, though potential cell-toxic effects must be considered. KLK6 appears essential for the neuronal cell function as it co-localizes with neurons (being unexpectedly localized intracellularly to the neuronal nucleus in the cytoplasm in a primary rat hippocampal neuronal cell model) and inhibits neuronal cell activity, differentiation and synaptic growth [63].

While the molecular mechanisms underlying the apparent proviral effect of KLK6 remain to be elucidated, several biologically plausible explanations may account for our findings. First, as an extracellular serine protease, KLK6 may modify the pericellular microenvironment through extracellular matrix remodeling or cleavage of cell surface proteins [64], thereby indirectly facilitating viral spread between adjacent cells, altering cell–cell communication, or modulating local inflammatory responses. Beyond extracellular protease activity, KLK6 may regulate gene expression, altering thousands of genes linked to neuronal immunity and connectivity, potentially contributing to neurodegeneration. Therefore, KLK6 may not only have properties of extracellular cleavage of substrates but might also have regulatory functions within the cell altering over 5,861 genes primarily related to neuronal immune regulation and cellular junctions [63]. KLK6 has been shown to activate protease-activated receptors (PAR-1 and PAR-2) [65], thereby influencing signaling pathways including NF-κB and MAPK, which are known to regulate inflammatory responses, cell survival, and HSV-1 replication.Finally, increased KLK6 expression may represent a host response to neuronal injury or cellular stress [47] that is subsequently exploited by HSV-1 to promote its replication, rather than reflecting a direct proviral function of KLK6 itself. KLK6 inhibits neurite outgrowth and adhesion via laminin cleavage, at least after neuronal injury in a rodent animal model [48], but also degrades myelin components, affecting axonal integrity [66]. While neurotoxic factors may induce KLK6, its role in limiting neuronal connections could restrict HSV-1 spread upon reactivation and reduce the harmful extent of inflammatory as well as infectious noxious agents to neighboring cells. Accordingly, elevated pTau observed in Alzheimer’s disease could, in part, represent a manifestation of heightened - potentially maladaptive - intracellular immune activation rather than exclusively a driver of neurodegenerative pathology [52]. In this scenario, the microtubule instability accompanying tau hyperphosphorylation could be conceptualized as a form of “raising the drawbridge” thereby impeding viral spread to neighboring cells via cell-to-cell connections, a process where extracellular cleavage of various structural targets by KLKs could be involved. These hypotheses remain speculative and require further mechanistic investigation before the in vitro findings can be extrapolated to the clinical setting.

While the clinical analyses demonstrate an association between the intrathecal anti-HSV immune response and CSF-KLK6 levels, the in vitro experiments provide independent functional evidence that KLK6 can influence HSV-1 replication under controlled experimental conditions. The precise mechanisms by which KLK6 facilitates HSV-1 replication remain to be elucidated. As an extracellular serine protease involved in extracellular matrix remodeling, neuronal plasticity, and inflammatory signaling, KLK6 may indirectly create a cellular microenvironment that favors HSV-1 replication or spread. Alternatively, increased KLK6 expression may represent a host stress response that is subsequently exploited by HSV-1. These mechanistic possibilities require further investigation before any clinical conclusions can be drawn.

## Limitations

Despite the large, well-characterized cohort, results need validation in independent studies. The mainly cross-sectional design limits conclusions on dynamic KLK or antibody-biomarker changes. Also, HSV-AI most likely reflect recurrent CNS antigen exposure including potential reactivation of latent herpesvirus within the brain. But the HSV-AI does not distinguish between latent infection, intermittent viral reactivation, persistent antigen exposure, or active lytic infection. Consequently, the observed associations should not be interpreted as evidence of ongoing viral replication within the CNS. Rather, the antibody index is interpreted as a marker of the intrathecal anti-HSV immune response that may reflect previous or recurrent exposure to HSV antigens within the CNS.

Future work should include CSF-KLK analysis in viral encephalitis as controls. It remains unclear whether KLK6 directly inhibits viral replication or affects amyloid homeostasis in vivo. Still, CSF-KLK6 may serve as a biomarker of HSV-1 reactivation and help clarify the viral role in AD. Observational studies during acute herpes infections with antiviral treatment could test whether CSF-KLK6 decreases and whether HSV-1 activity and KLK6 jointly influence AD biomarkers.

## Conclusion

KLK6 appears to participate in productive HSV-1 infection in vitro and is associated with the intrathecal anti-HSV immune response in patients with Alzheimer’s disease. Together with its association with established AD biomarkers, these findings support KLK6 as a candidate biomarker linking herpesvirus-associated immune responses with neurodegenerative processes. Further studies are warranted to determine its mechanistic role and to evaluate its potential for assessing the contribution of alphaherpesviruses to Alzheimer’s disease and other neurodegenerative disorders.

## Supplementary Information

Supplementary Material 1.

Supplementary Material 2.

## Data Availability

Pseudonymized clinical data can be received in accordance with European data protection legislation after the conclusion of a data transfer agreement on relevant grounds. Independently, laboratory data can be obtained upon reasonable request.

## Abbreviations

Aβ: Amyloid-beta
Aβ40: Amyloid-beta 1–40
Aβ42: Amyloid-beta 1–42
Aβ42/Aβ40 ratio: Ratio of amyloid-beta 1–42 to amyloid-beta 1–40
AD: Alzheimer’s disease
AI: Antibody index
ALP: Autophagy-lysosomal pathway
ANOVA: Analysis of variance
APOE: Apolipoprotein E
APP: Amyloid precursor protein
AU: Arbitrary units
BafA1: Bafilomycin A1
BCA: Bicinchoninic acid
BSA: Bovine serum albumin
BSL-2: Biosafety level 2
CDR: Clinical Dementia Rating scale
cDNA: Complementary DNA
CE: European conformity
CHX: Cycloheximide
CMV: Cytomegalovirus
CMV-AI: Anti-CMV IgG CSF-to-serum antibody index
CNS: Central nervous system
CONICYT: National Commission for Scientific and Technological Research (Chile)
CSF: Cerebrospinal fluid
CV: Coefficient of variation
DAMPs: Danger-associated molecular patterns
DAPI: 4′,6-Diamidino-2-phenylindole
DMEM: Dulbecco’s Modified Eagle Medium
DMSO: Dimethyl sulfoxide
DNA: Deoxyribonucleic acid
EAR: Expressed Alu repeats
ELISA: Enzyme-linked immunosorbent assay
FBS: Fetal bovine serum
hpi: Hours post infection
HSV+: HSV-1 seropositive
HSV-: HSV-1 seronegative
HSV-1: Herpes simplex virus type 1
HSV-AI: Anti-HSV-1/2 IgG CSF-to-serum antibody index
IFI: Indirect immunofluorescence
IgG: Immunoglobulin G
IgM: Immunoglobulin M
KLK: Kallikrein-related peptidase
KLK1-12: Kallikrein-related peptidases 1–12
KS: Kolmogorov Smirnov
LP: Lumbar puncture
MDM2: Mouse double minute 2 homolog
MG132: Carbobenzoxy-Leu-Leu-Leu-al
MMSE: Mini-Mental State Examination
MOI: Multiplicity of infection
mRNA: Messenger RNA
NC: Normal control
NP-40: Nonidet P-40
OE: Overexpression
PBS: Phosphate-buffered saline
PCR: Polymerase chain reaction
pTau: Phosphorylated tau
pTau181: Phosphorylated tau at threonine 181
Q_alb_: CSF-to-serum albumin ratio
Q_lim_: Upper discrimination line of the reference range
Q_spec_: Specific IgG CSF-to-serum quotient
Q_IgG_: IgG CSF-to-serum quotient
RNA: Ribonucleic acid
RT-qPCR: Reverse transcription quantitative polymerase chain reaction
SD: Standard deviation
SDS: Sodium dodecyl sulfate
SDS-PAGE: Sodium dodecyl sulfate polyacrylamide gel electrophoresis
siKLK6: Small interfering RNA targeting KLK6
siNT: Non-targeting small interfering RNA
SPSS: Statistical Package for the Social Sciences
TCID50: 50% Tissue culture infectious dose
TEMED: Tetramethylethylenediamine
tTau: Total tau
UPS: Ubiquitin-proteasome system
UV: Ultraviolet
VZV: Varicella zoster virus

## References

[1] Jack CR Jr., Bennett DA, Blennow K, Carrillo MC, Dunn B, Haeberlein SB, et al. NIA-AA Research Framework: Toward a biological definition of Alzheimer’s disease. Alzheimers Dement. 2018;14(4):535–62. 10.1016/j.jalz.2018.02.018 PMC5958625 29653606

[2] Campion D, Dumanchin C, Hannequin D, Dubois B, Belliard S, Puel M, et al. Early-onset autosomal dominant Alzheimer disease: prevalence, genetic heterogeneity, and mutation spectrum. Am J Hum Genet. 1999;65(3):664–70. 10.1086/302553 PMC1377972 10441572

[3] Goldhardt O, Warnhoff I, Yakushev I, Begcevic I, Forstl H, Magdolen V, et al. Kallikrein-related peptidases 6 and 10 are elevated in cerebrospinal fluid of patients with Alzheimer’s disease and associated with CSF-TAU and FDG-PET. Transl Neurodegener. 2019;8:25. 10.1186/s40035-019-0168-6 PMC6712703 31467673

[4] Grimmer T, Goldhardt O, Yakushev I, Ortner M, Sorg C, Diehl-Schmid J, et al. Associations of Neprilysin Activity in CSF with Biomarkers for Alzheimer’s Disease. Neurodegener Dis. 2019;19(1):43–50. 10.1159/000500811 31266021

[5] Venegas C, Heneka MT. Danger-associated molecular patterns in Alzheimer’s disease. J Leukoc Biol. 2016;101(1):87–98. 10.1189/jlb.3MR0416-204R 28049142

[6] Hillen H. The Beta Amyloid Dysfunction (BAD) Hypothesis for Alzheimer’s Disease. Front Neurosci. 2019;13:1154. 10.3389/fnins.2019.01154 PMC6853841 31787864

[7] Muller EG, Edwin TH, Stokke C, Navelsaker SS, Babovic A, Bogdanovic N, et al. Amyloid-beta PET-Correlation with cerebrospinal fluid biomarkers and prediction of Alzheimer s disease diagnosis in a memory clinic. PLoS ONE. 2019;14(8):e0221365. 10.1371/journal.pone.0221365 PMC6701762 31430334

[8] Kametani F, Hasegawa M. Reconsideration of Amyloid Hypothesis and Tau Hypothesis in Alzheimer’s Disease. Front Neurosci. 2018;12:25. 10.3389/fnins.2018.00025 PMC5797629 29440986

[9] Arnsten AFT, Datta D, Del Tredici K, Braak H. Hypothesis: Tau pathology is an initiating factor in sporadic Alzheimer’s disease. Alzheimers Dement. 2021;17(1):115–24. 10.1002/alz.12192 PMC7983919 33075193

[10] Brothers HM, Gosztyla ML, Robinson SR. The Physiological Roles of Amyloid-beta Peptide Hint at New Ways to Treat Alzheimer’s Disease. Front Aging Neurosci. 2018;10:118. 10.3389/fnagi.2018.00118 PMC5996906 29922148

[11] Hellenbrand W, Thierfelder W, Muller-Pebody B, Hamouda O, Breuer T. Seroprevalence of herpes simplex virus type 1 (HSV-1) and type 2 (HSV-2) in former East and West Germany, 1997–1998. Eur J Clin Microbiol Infect Dis. 2005;24(2):131–5. 10.1007/s10096-005-1286-x 15692814

[12] De Chiara G, Piacentini R, Fabiani M, Mastrodonato A, Marcocci ME, Limongi D, et al. Recurrent herpes simplex virus-1 infection induces hallmarks of neurodegeneration and cognitive deficits in mice. PLoS Pathog. 2019;15(3):e1007617. 10.1371/journal.ppat.1007617 PMC6417650 30870531

[13] Jamieson GA, Maitland NJ, Wilcock GK, Yates CM, Itzhaki RF. Herpes simplex virus type 1 DNA is present in specific regions of brain from aged people with and without senile dementia of the Alzheimer type. J Pathol. 1992;167(4):365–8. 10.1002/path.1711670403 1328575

[14] Eimer WA, Vijaya Kumar DK, Navalpur Shanmugam NK, Rodriguez AS, Mitchell T, Washicosky KJ, et al. Alzheimer’s Disease-Associated beta-Amyloid Is Rapidly Seeded by Herpesviridae to Protect against Brain Infection. Neuron. 2018;99(1):56–e633. 10.1016/j.neuron.2018.06.030 PMC6075814 30001512

[15] Bourgade K, Garneau H, Giroux G, Le Page AY, Bocti C, Dupuis G, et al. β-Amyloid peptides display protective activity against the human Alzheimer’s disease-associated herpes simplex virus-1. Biogerontology. 2015;16(1):85–98. 10.1007/s10522-014-9538-8 25376108

[16] Bourgade K, Le Page A, Bocti C, Witkowski JM, Dupuis G, Frost EH, et al. Protective Effect of Amyloid-β Peptides Against Herpes Simplex Virus-1 Infection in a Neuronal Cell Culture Model. J Alzheimer’s Disease. 2016;50(4):1227–41. 10.3233/JAD-150652 26836158

[17] Wozniak MA, Frost AL, Itzhaki RF. Alzheimer’s disease-specific tau phosphorylation is induced by herpes simplex virus type 1. J Alzheimers Dis. 2009;16(2):341–50. 10.3233/JAD-2009-0963 19221424

[18] Lerchundi R, Neira R, Valdivia S, Vio K, Concha MI, Zambrano A, et al. Tau cleavage at D421 by caspase-3 is induced in neurons and astrocytes infected with herpes simplex virus type 1. J Alzheimers Dis. 2011;23(3):513–20. 10.3233/JAD-2010-101386 21098975

[19] Zambrano A, Solis L, Salvadores N, Cortes M, Lerchundi R, Otth C. Neuronal cytoskeletal dynamic modification and neurodegeneration induced by infection with herpes simplex virus type 1. J Alzheimers Dis. 2008;14(3):259–69. 10.3233/jad-2008-14301 18599953

[20] Goldhardt O, Freiberger R, Dreyer T, Willner L, Yakushev I, Ortner M, et al. Herpes simplex virus alters Alzheimer’s disease biomarkers - A hypothesis paper. Alzheimers Dement. 2023;19(5):2117–34. 10.1002/alz.12834 36396609

[21] Readhead B, Haure-Mirande JV, Funk CC, Richards MA, Shannon P, Haroutunian V, et al. Multiscale Analysis of Independent Alzheimer’s Cohorts Finds Disruption of Molecular, Genetic, and Clinical Networks by Human Herpesvirus. Neuron. 2018;99(1):64–82. e7. 10.1016/j.neuron.2018.05.023 PMC6551233 29937276

[22] Allnutt MA, Johnson K, Bennett DA, Connor SM, Troncoso JC, Pletnikova O, et al. Human Herpesvirus 6 Detection in Alzheimer’s Disease Cases and Controls across Multiple Cohorts. Neuron. 2020;105(6):1027–35. e2. 10.1016/j.neuron.2019.12.031 PMC7182308 31983538

[23] Chorlton SD. Reanalysis of Alzheimer’s brain sequencing data reveals absence of purported HHV6A and HHV7. J Bioinform Comput Biol. 2020;18(1):2050012. 10.1142/S0219720020500122 32336252

[24] Jamieson GA, Maitland NJ, Wilcock GK, Craske J, Itzhaki RF. Latent herpes simplex virus type 1 in normal and Alzheimer’s disease brains. J Med Virol. 1991;33(4):224–7. 10.1002/jmv.1890330403 1649907

[25] Olsson J, Lovheim H, Honkala E, Karhunen PJ, Elgh F, Kok EH. HSV presence in brains of individuals without dementia: the TASTY brain series. Dis Model Mech. 2016;9(11):1349–55. 10.1242/dmm.026674 PMC5117234 27664135

[26] Gosztyla ML, Brothers HM, Robinson SR. Alzheimer’s Amyloid-beta is an Antimicrobial Peptide: A Review of the Evidence. J Alzheimers Dis. 2018;62(4):1495–506. 10.3233/JAD-171133 29504537

[27] Debela M, Beaufort N, Magdolen V, Schechter NM, Craik CS, Schmitt M, et al. Structures and specificity of the human kallikrein-related peptidases KLK 4, 5, 6, and 7. Biol Chem. 2008;389(6):623–32. 10.1515/BC.2008.075 18627343

[28] Shimizu-Okabe C, Yousef GM, Diamandis EP, Yoshida S, Shiosaka S, Fahnestock M. Expression of the kallikrein gene family in normal and Alzheimer’s disease brain. NeuroReport. 2001;12(12):2747–51. 10.1097/00001756-200108280-00031 11522960

[29] Patra K, Soosaipillai A, Sando SB, Lauridsen C, Berge G, Moller I, et al. Assessment of kallikrein 6 as a cross-sectional and longitudinal biomarker for Alzheimer’s disease. Alzheimers Res Ther. 2018;10(1):9. 10.1186/s13195-018-0336-4 PMC5789599 29378650

[30] Mella C, Figueroa CD, Otth C, Ehrenfeld P. Involvement of Kallikrein-Related Peptidases in Nervous System Disorders. Front Cell Neurosci. 2020;14:166. 10.3389/fncel.2020.00166 PMC7324807 32655372

[31] Bin L, Kim BE, Hall CF, Leach SM, Leung DY. Inhibition of transcription factor specificity protein 1 alters the gene expression profile of keratinocytes leading to upregulation of kallikrein-related peptidases and thymic stromal lymphopoietin. J Invest Dermatol. 2011;131(11):2213–22. 10.1038/jid.2011.202 PMC3193562 21753780

[32] Sodroski CN, Oh HS, Chou SF, Knipe DM. Sp1 facilitates continued HSV-1 gene expression in the absence of key viral transactivators. mBio. 2024;15(3):e0347923. 10.1128/mbio.03479-23 PMC10936440 38349188

[33] Tommasi C, Rogerson C, Depledge DP, Jones M, Naeem AS, Venturini C, et al. Kallikrein-Mediated Cytokeratin 10 Degradation Is Required for Varicella Zoster Virus Propagation in Skin. J Invest Dermatol. 2020;140(4):774–84. e11. 10.1016/j.jid.2019.08.448 31626786

[34] McKhann GM, Knopman DS, Chertkow H, Hyman BT, Jack CR Jr, Kawas CH, et al. The diagnosis of dementia due to Alzheimer’s disease: Recommendations from the National Institute on Aging-Alzheimer’s Association workgroups on diagnostic guidelines for Alzheimer’s disease. Alzheimer’s Dement. 2011;7(3):263–9. 10.1016/j.jalz.2011.03.005 PMC3312024 21514250

[35] Diamandis EP, Yousef GM, Soosaipillai AR, Grass L, Porter A, Little S, et al. Immunofluorometric assay of human kallikrein 6 (zyme/protease M/neurosin) and preliminary clinical applications. Clin Biochem. 2000;33(5):369–75. 10.1016/s0009-9120(00)00145-4 11018688

[36] Kishi T, Grass L, Soosaipillai A, Shimizu-Okabe C, Diamandis EP. Human Kallikrein 8: Immunoassay Development and Identification in Tissue Extracts and Biological Fluids. Clin Chem. 2003;49(1):87–96. 10.1373/49.1.87 12507964

[37] Luo LY, Grass L, Howarth DJ, Thibault P, Ong H, Diamandis EP. Immunofluorometric assay of human kallikrein 10 and its identification in biological fluids and tissues. Clin Chem. 2001;47(2):237–46. 11159772

[38] Zarghooni M, Soosaipillai A, Grass L, Scorilas A, Mirazimi N, Diamandis EP. Decreased concentration of human kallikrein 6 in brain extracts of Alzheimer’s disease patients. Clin Biochem. 2002;35(3):225–31. 10.1016/s0009-9120(02)00292-8 12074831

[39] Shipley MM, Mangold CA, Szpara ML. Differentiation of the SH-SY5Y Human Neuroblastoma Cell Line. J Vis Exp. 2016;108:53193. 10.3791/53193 PMC4828168 26967710

[40] Wiltfang J, Esselmann H, Bibl M, Hull M, Hampel H, Kessler H, et al. Amyloid beta peptide ratio 42/40 but not A beta 42 correlates with phospho-Tau in patients with low- and high-CSF A beta 40 load. J Neurochem. 2007;101(4):1053–9. 10.1111/j.1471-4159.2006.04404.x 17254013

[41] Pandey R, Zhou M, Chen Y, Darmoul D, Kisiel CC, Nfonsam VN et al. Molecular Pathways Associated with Kallikrein 6 Overexpression in Colorectal Cancer. Genes (Basel). 2021;12(5):749. 10.3390/genes12050749 PMC8157155 34065672

[42] Dahlgren KN, Manelli AM, Stine WB, Baker LK, Krafft GA, LaDu MJ. Oligomeric and Fibrillar Species of Amyloid-β Peptides Differentially Affect Neuronal Viability*. J Biol Chem. 2002;277(35):32046–53. 10.1074/jbc.M201750200 12058030

[43] Lacham-Hartman S, Oren O, Rabinovitch M, Papo N. Neuroprotective functions of the APPI domain of amyloid precursor protein (APP): Reducing KLK6-mediated APP cleavage and Aβ42 formation and aggregation. Int J Biol Macromol. 2025:146066. 10.1016/j.ijbiomac.2025.146066 40680950

[44] Little SP, Dixon EP, Norris F, Buckley W, Becker GW, Johnson M, et al. Zyme, a novel and potentially amyloidogenic enzyme cDNA isolated from Alzheimer’s disease brain. J Biol Chem. 1997;272(40):25135–42. 10.1074/jbc.272.40.25135 9312124

[45] Stefanini AC, da Cunha BR, Henrique T, Tajara EH. Involvement of Kallikrein-Related Peptidases in Normal and Pathologic Processes. Dis Markers. 2015;2015:946572. 10.1155/2015/946572 PMC4689925 26783378

[46] Prassas I, Eissa A, Poda G, Diamandis EP. Unleashing the therapeutic potential of human kallikrein-related serine proteases. Nat Rev Drug Discovery. 2015;14(3):183–202. 10.1038/nrd4534 25698643

[47] Scarisbrick IA, Radulovic M, Burda JE, Larson N, Blaber SI, Giannini C, et al. Kallikrein 6 is a novel molecular trigger of reactive astrogliosis. Biol Chem. 2012;393(5):355–67. 10.1515/hsz-2011-0241 PMC3335747 22505518

[48] Scarisbrick IA, Sabharwal P, Cruz H, Larsen N, Vandell AG, Blaber SI, et al. Dynamic role of kallikrein 6 in traumatic spinal cord injury. Eur J Neurosci. 2006;24(5):1457–69. 10.1111/j.1460-9568.2006.05021.x 16987227

[49] Kumar A, Fontana IC, Nordberg A. Reactive astrogliosis: A friend or foe in the pathogenesis of Alzheimer’s disease. J Neurochem. 2023;164(3):309–24. 10.1111/jnc.15565 34931315

[50] Leng F, Edison P. Neuroinflammation and microglial activation in Alzheimer disease: where do we go from here? Nat Reviews Neurol. 2021;17(3):157–72. 10.1038/s41582-020-00435-y 33318676

[51] Roizman B, Whitley RJ. An inquiry into the molecular basis of HSV latency and reactivation. Annu Rev Microbiol. 2013;67:355–74. 10.1146/annurev-micro-092412-155654 24024635

[52] Eimer WA, Rodriguez AS, DeFao MT, Ehricke S, Park J, Vijaya Kumar DK et al. Phosphorylated tau exhibits antimicrobial activity capable of neutralizing herpes simplex virus 1 infectivity in human neurons.*Nat Neurosci*29, 604–616 (2026). 10.1038/s41593-025-02157-0 PMC12971498 41408481

[53] Bayés A, Tsetsenis T, Ventura S, Vendrell J, Aviles FX, Sotiropoulou G. Human kallikrein 6 activity is regulated via an autoproteolytic mechanism of activation/inactivation. Biol Chem. 2004;385(6):517–24. 10.1515/BC.2004.061 15255184

[54] Kwon YT, Ciechanover A. The Ubiquitin Code in the Ubiquitin-Proteasome System and Autophagy. Trends Biochem Sci. 2017;42(11):873–86. 10.1016/j.tibs.2017.09.002 28947091

[55] Albornoz N, Bustamante H, Soza A, Burgos P. Cellular Responses to Proteasome Inhibition: Molecular Mechanisms and Beyond. Int J Mol Sci. 2019;20(14):3379. 10.3390/ijms20143379 PMC6678303 31295808

[56] Orvedahl A, Alexander D, Tallóczy Z, Sun Q, Wei Y, Zhang W, et al. HSV-1 ICP34.5 Confers Neurovirulence by Targeting the Beclin 1 Autophagy Protein. Cell Host Microbe. 2007;1(1):23–35. 10.1016/j.chom.2006.12.001 18005679

[57] Gobeil PAM, Leib DA. Herpes Simplex Virus γ34.5 Interferes with Autophagosome Maturation and Antigen Presentation in Dendritic Cells. mBio. 2012;3(5). 10.1128/mbio.00267-12. PMC3470650 23073763

[58] Nixon RA. Amyloid precursor protein and endosomal-lysosomal dysfunction in Alzheimer’s disease: inseparable partners in a multifactorial disease. FASEB J. 2017;31(7):2729–43. 10.1096/fj.201700359 PMC6137496 28663518

[59] Pino-Belmar C, Aguilar R, Valenzuela-Nieto GE, Cavieres VA, Cerda-Troncoso C, Navarrete VC, et al. An Intrinsic Host Defense against HSV-1 Relies on the Activation of Xenophagy with the Active Clearance of Autophagic Receptors. Cells. 2024;13(15):1256. 10.3390/cells13151256 PMC11311385 39120287

[60] Maruzuru Y, Fujii H, Oyama M, Kozuka-Hata H, Kato A, Kawaguchi Y. Roles of p53 in herpes simplex virus 1 replication. J Virol. 2013;87(16):9323–32. 10.1128/JVI.01581-13 PMC3754062 23785201

[61] Herring A, Münster Y, Akkaya T, Moghaddam S, Deinsberger K, Meyer J, et al. Kallikrein-8 inhibition attenuates Alzheimer’s disease pathology in mice. Alzheimer’s Dement. 2016;12(12):1273–87. 10.1016/j.jalz.2016.05.006 27327541

[62] Keyvani K, Münster Y, Kurapati NK, Rubach S, Schönborn A, Kocakavuk E, et al. Higher levels of kallikrein-8 in female brain may increase the risk for Alzheimer’s disease. Brain Pathol. 2018;28(6):947–64. 10.1111/bpa.12599 PMC8028555 29505099

[63] Yuan L, Zou D, Yang X, Chen X, Lu Y, Zhang A, et al. Proteomics and functional study reveal kallikrein-6 enhances communicating hydrocephalus. Clin Proteomics. 2021;18(1):30. 10.1186/s12014-021-09335-9 PMC8903716 34915845

[64] Srinivasan S, Kryza T, Batra J, Clements J. Remodelling of the tumour microenvironment by the kallikrein-related peptidases. Nat Rev Cancer. 2022;22(4):223–38. 10.1038/s41568-021-00436-z 35102281

[65] Yoon H, Radulovic M, Wu J, Blaber SI, Blaber M, Fehlings MG, et al. Kallikrein 6 signals through PAR1 and PAR2 to promote neuron injury and exacerbate glutamate neurotoxicity. J Neurochem. 2013;127(2):283–98. 10.1111/jnc.12293 PMC4097186 23647384

[66] Burda JE, Radulovic M, Yoon H, Scarisbrick IA. Critical role for PAR1 in kallikrein 6-mediated oligodendrogliopathy. Glia. 2013;61(9):1456–70. 10.1002/glia.22534 PMC4097182 23832758

[67] Marullo M, Zuccato C, Mariotti C, Lahiri N, Tabrizi SJ, Di Donato S, et al. Expressed Alu repeats as a novel, reliable tool for normalization of real-time quantitative RT-PCR data. Genome Biol. 2010;11(1):R9. 10.1186/gb-2010-11-1-r9 PMC2847721 20109193

[68] Verzosa AL, McGeever LA, Bhark SJ, Delgado T, Salazar N, Sanchez EL. Herpes Simplex Virus 1Infection of Neuronal and Non-Neuronal Cells Elicits Specific Innate ImmuneResponses and Immune Evasion Mechanisms. Front Immunol. 2021;12:644664. 10.3389/fimmu.2021.644664 PMC8201405 34135889

[69] Schelhaas M, JansenM, Haase I, Knebel-Morsdorf D. Herpes simplex virus type 1 exhibits a tropismfor basal entry in polarized epithelial cells. J Gen Virol. 2003;84(Pt9):2473-84. 10.1099/vir.0.19226-0 12917468

[70] Kropp KA, Sun G,Viejo-Borbolla A. Colonization of peripheral ganglia by herpes simplex virustype 1 and 2. Curr Opin Virol. 2023;60:101333. 10.1016/j.coviro.2023.101333 37267706

