# Supplementary material for: Herpes simplex virus and kallikrein-related peptidases – a functional connection in Alzheimer‘s disease
Source: Alzheimers Res Ther. 2026 Sep 24;18:212. doi: 10.1186/s13195-026-02204-3 (PMC13615609; doi:10.1186/s13195-026-02204-3)
Supplement: Supplementary file 1 [file 13195_2026_2204_MOESM1_ESM.docx]

**Additional file 1**

of the manuscript ‘Herpes simplex virus and Kallikrein-related peptidases – a functional connection in Alzheimer‘s disease’

**Figures**

Additional file 1 **Figure 1:** Correlations among AD biomarkers and KLKs in the CSF stratified by HSV-1 seropositivity

**Legend to** Additional file 1 **Figure 1**: Spearman Rho correlation of all 114 patients with AD or stratified by HSV-seropositivity (24 seronegative, 90 seropositive) controlled for multiple testing with Bonferroni method (1A: factor 10, 1B: factor 6). Positive (blue) or negative (orange) correlation coefficients are indicated, and display in circles if p-values ≤ 0.05.

**Additional file 1 Figure 2: CSF-KLK6 indicates intrathecal HSV-1 activity**

**Legend to Additional file 1 Figure 2**: Patients were stratified into HSV−, HSV+AI<1.5, and HSV+AI≥1.5 groups. Among CSF-KLK6–8 and KLK10, only KLK6 showed a trend toward enrichment in HSV+AI≥1.5 (p=0.059), while KLK7 was numerically higher without significance (CSF-KLK7: p=0.218, CSF-KLK8: p=0.954, KLK10: p=0.589). No significant differences were observed for KLK6–8 or KLK10 with CMV serostatus or CMV-AI stratification. After using Kruskal-Wallis-Test, subgroups comparison were calculated with post hoc Mann Whitney U test. KLK6 was significantly higher in the HSV+ AI≥1.5 group, if compared to HSV- and HSV+ AI<1.5 patients together, or if compared to HSV- patients alone.

**Additional file 1 Figure 3: Correlations between alterations of CSF AD biomarkers and KLK at baseline**

**Legend to Additional file 1 Figure 3**: (A) Cross-sectional moderator analyses with Seither CSF-pTau181 or CSF-tTau as dependent variables, HSV-AI as independent variable and CSF-KLK6 as moderator variable showing significant models with direct an indirect significant effects. (B) Longitudinal alterations of CSF AD biomarkers per year correlated with CSF-KLK6 levels at first CSF assessment (baseline).

**Additional file 1 Figiure 4: Expression of basal and inducible kallikreins during productive HSV-1 infection on HaCaT cells.**

**Legend to Additional file 1 Figure 4**: Western blot analyses show (A) KLK1 and KLK7 as inducibles proteins expression, constitutive protein (β-Actin) and their respective densitrometric analysis during HSV-1 infection (B-C). (D) KLK5, KLK7 and KLK10 as basal proteins expression, constitutive protein β-Actin and their densitrometric analysis (E-F). (G) KLRT10 and p53 proteins expression during HSV-1 infection . All kinetics were performed in uninfected HaCaT cells (Mock or M) or infected with HSV-1 at multiplicity of infection (MOI = 10).

**Additional file 1 Tables**

**Additional file 1 Table 1: Stratification of patient cohort by intrathecal CMV activity**

| total n=101^$^ | AD CMV-  n= 64 | AD CMV+ AI < 1.5  n = 32 | AD CMV+ AI ≥ 1.5  n = 5 | Kruskal Wallis test |
| --- | --- | --- | --- | --- |
| CSF AD biomarkers | | | | |
| Aβ42 [ng/l] | 464 ± 120.6 (236-797) | 502 ± 165.9 (232-909) | 493 ± 100.3 (409-622) | 0.508 |
| Aβ40 [ng/l] x 10E3 | 12672 ± 3656.1 (5900-21900) | 14732 ± 5120.1 (5060-26423) | 13442 ± 4413.5 (9128-20733) | 0.187 |
| Aβ42/Aβ40 ratio | 0.04 ± 0.01 (0.02-0.05) | 0.04 ± 0.01 (0.02-0.06) | 0.04 ± 0.01 (0.03-0.05) | 0.527 |
| pTau [ng/l] | 87 ± 40 (36-229) | 110 ± 53 (44-231) | 82 ± 40 (46-145) | 0.145 |
| tTau [ng/l] | 736 ± 387 (218-2165) | 986 ± 623 (238-3047) | 605 ± 311 (314-1099) | 0.132 |
|  |  |  |  |  |
| CSF kallikrein-related peptidases | | | | |
| KLK6 [ng/ml] | 360 ± 113.3 (198-850) | 401 ± 166.0 (206-930) | 372 ± 126.6 (256-585) | 0.608 |
| KLK7 [ng/ml] | 2.0 ± 0.84 (0.8-4.9) | 2.2 ± 1.09 (1.0-61.) | 2.2 ± 1.20 (1.2-4.0) | 0.975 |
| KLK8 [ng/ml] | 0.05 ± 0.017 (0.02-0.09) | 0.05 ± 0.023 (0.02-0.12) | 0.05 ± 0.021 (0.03-0.08) | 0.728 |
| KLK10 [ng/ml] | 0.51 ± 0.239 (0.07-1.06) | 0.54 ± 0.292 (0.10-34) | S-58 ± 0.229 (0.36-0.96) | 0.873 |
|  |  |  |  |  |
| Demographic data | | | | |
| Age [years] | 68 ± 8.4 (48-83) | 71 ± 7.9 (54-87) | 72 ± 10.5 (55-83) | 0.259 |
| Sex [f/m] | 33 / 31 | 18 / 14 | 2 / 3 |  |
| MMSE | 24 ± 3.5 (13-30) | 25 ± 3.4 (17-30) | 23 ± 6.5 (14-28) | 0.182 |
| Global CDR  [0/0.5/1/2/3] | 0.8 ± 0.36 (0.5-2) | 0.7 ± 0.25 (0.5-1) | 0.6 ± 0.22 (0.5-1) | 0.224 |
| APOE ε4 alleles  [0/1/2] | 26 / 23 / 14 | 10 / 16 / 5 | 1 / 4 / 0 |  |
| CSF/serum albumin ratio | 6.9 ± 2.68 (3.3-17.2) | 7.5 ± 4.17 (4.1-26.5) | 8.6 ± 2.81 (5.9-13.3) | 0.283 |
| CSF/serum unspecific IgG ratio | 3.1 ± 1.21 (1.3-7.9) | 3.5 ± 2.22 (1.8-13.5) | 3.9 ± 1.53 (2.5-6.4) | 0.413 |

**Legend to Additional file 1 Table 1:** After stratification of the patient cohort according to intrathecal CMV activity, mean differences were tested between all groups using ANOVA or asymptotic Pearson chi square test (asterisks) dependent on the variable‘s metric. In 13 CMV-seropositive patients ($) CMV-AI could not be determined within range (i.e below 0.5). AD: Alzheimer’s disease, Aβ42 or Aβ40: amyloid 1-42 or amyloid 1-40, ApoE: Apolipoprotein E, AI-IgGHSV-1/2: CSF to serum anti-HSV-1/2-IgG antibody index, CDR: Clinical Dementia Rating scale, CSF: cerebrospinal fluid, HSV-1: herpes simplex virus type 1, KLK: kallikrein-related peptidase, MMSE: Mini-Mental state examination, MWU: Mann-Whitney-U test, pTau181: phospho Tau 181, tTau: total Tau.

Additional file 1 Table 2: Regression diagnostics and model robustness

| Diagnostic | Results | Interpretation |
| --- | --- | --- |
| Multicollinearity (centered model) | VIF:  HSV-AI = 1.03  Aβ42/40 ratio = 1.04  Interaction = 1.06 | no relevant multicollinearity after centering |
| Cook&apos;s distance | Maximum = 0.233;  4 observations > 4/n (0.05) | a small number of moderately influential observations; no single observation dominated the model |
| Leverage | Maximum = 0.418;  7 observations > 2p/n | moderately increased leverage, consistent with biological variability |
| Externally studentized residuals | 2 observations with \|rstudent\| > 3 | only two potential residual outliers |
| Residual distribution | Omnibus p < 0.001; Jarque–Bera p < 0.001 | residuals deviated from normality, mainly due to right-skewed KLK6 values |

Legend to Supplement Table 2: Regression diagnostics of the multivariable model predicting CSF-KLK6. Continuous predictors were mean-centered before generating the interaction term. Model robustness was assessed by VIFs, Cook&apos;s distance, leverage, externally studentized residuals, and residual normality tests.
