# Supplementary material for: Herpes simplex virus and kallikrein-related peptidases – a functional connection in Alzheimer‘s disease
Source: Alzheimers Res Ther. 2026 Sep 24;18:212. doi: 10.1186/s13195-026-02204-3 (PMC13615609; doi:10.1186/s13195-026-02204-3)
Supplement: Supplementary file 2 [file 13195_2026_2204_MOESM2_ESM.docx]

KLK6 indicates HSV-1 activity in AD – Goldhardt and Mella et al. 2026 – SUPPLEMENT

**Additional file 2**

of the manuscript ‘Herpes simplex virus and Kallikrein-related peptidases – a functional connection in Alzheimer‘s disease’

| **FIGURE 4.A**  **H4 cells** |  | | |
| --- | --- | --- | --- |
| **N1** | **N2** | **N3** | **ICP5** |
|  |  |   **SELECTED FOR FIGURE** |  |
| **N1** | **N2** | **ICP0** | |
|   **SELECTED FOR FIGURE** |  |  |  |
| **N1** | **N2** | **N3** | **ICP27** |
|   **SELECTED FOR FIGURE** |  |  |  |
| **N1** | **N2** | **N3** | **ICP8** |
|  |   **SELECTED FOR FIGURE** |  |  |
| **N1** | **N2** | **N3** | **ß-ACTIN** |
|   **SELECTED FOR FIGURE** |  |  |  |

| **FIGURE 4.C**  **H4 cells** |  | | |
| --- | --- | --- | --- |
| **N1** | **N2** | **N3** | **KLK6** |
|   **Used for densitometric quantification** |   **Used for densitometric quantification** |   **Ignore the last line (sample from another set)**  **Used for densitometric quantification**  **SELECTED FOR FIGURE** |  |
| **N1** | **N2** | **N3** | **ICP8** |
|  |  |   **SELECTED FOR FIGURE** |  |
| **N1** | **N2** | **N3** | **ß-ACTIN** |
|   **Used for densitometric quantification** |   **Used for densitometric quantification** |   **Ignore the last line (sample from another set)**  **Used for densitometric quantification**  **SELECTED FOR FIGURE** |  |

| **FIGURE 4.E**  **H4 cells** |  | | |
| --- | --- | --- | --- |
| **N1** | **N2** | **N3** | **KLK6** |
|   **SELECTED FOR FIGURE** |  |  |  |
| **N1** | **N2** | **ICP8** | |
|   **SELECTED FOR FIGURE** |  |  |  |
| **N1** | **N2** | **N3** | **ß-ACTIN** |
|  |  |   **SELECTED FOR FIGURE** |  |

| **FIGURE 4.H**  **SHSY5Y cells** |  | | | | |
| --- | --- | --- | --- | --- | --- |
| **N1** | **N2** | **N3** | **N4** | | **KLK6** |
|   **Used for densitometric quantification** |   **Used for densitometric quantification** |   **consider only the first 7 lines**  **Used for densitometric quantification**  **SELECTED FOR FIGURE** |  | |  |
| **N1** | **N2** | **N3** | | **N4** | **ICP8** |
|  |  |   **consider only the first 6 lines**  **SELECTED FOR FIGURE** | |  |  |
| **N1** | **N2** | **N3** | **N4** | | **ß-ACTIN** |
|   **Used for densitometric quantification** |   **Used for densitometric quantification** | **consider only the first 6 lines**  **Used for densitometric quantification**  **SELECTED FOR FIGURE** |  | |  |

| **FIGURA 5.A**  **HaCaT cells** |  | | |
| --- | --- | --- | --- |
| **N1** | **N2** | **N3** | **ICP5** |
|   **Used for densitometric quantification** |   **Used for densitometric quantification** |   **Used for densitometric quantification**  **SELECTED FOR FIGURE** |  |
| **N1** | **N2** | **N3** | **ICP0** |
|   **Used for densitometric quantification**  **SELECTED FOR FIGURE** |   **Used for densitometric quantification** |   **Ignore the last line (H4 cells 24 hpi)**  **Usedfor densitometric quantification** |  |
| **N1** | **N2** | **N3** | **ICP27** |
|   **Used for densitometric quantification** |   **Used for densitometric quantification SELECTED FOR FIGURE** |   **Used for densitometric quantification** |  |
| **N1** | **N2** | **N3** | **ICP8** |
|   **Used for densitometric quantification** |   **Used for densitometric quantification SELECTED FOR FIGURE** |   **Ignore the last line (H4 cells 24 hpi)**  **Used for densitometric quantification** |  |
| **N1** | **N2** | **N3** | **ß- ACTIN** |
|   **Ignore the last line (This loading control was carried out separately alongside a sample from another set)**  **Used for densitometric quantification** |   **Used for densitometric quantification SELECTED FOR FIGURE** |   **Ignore the last line (H4 cells 24 hpi)**  **Used for densitometric quantification** |  |
| **N3 and N4** | | **N5 and N6** | **HSV-1 Total Proteins**  **(Full membrane)** |
|   **N3 (left) N4 (right)**  **N3 SELECTED FOR FIGURE** | |   **N5 (left) N6 (right)** |  |

| **FIGURA 5.F**  **HaCaT cells** |  | | |
| --- | --- | --- | --- |
| **N1** | **N2** | **N3** | **KLK6** |
|   **Ignore the last line**  **Used for densitometric quantification**  **SELECTED FOR FIGURE** |   **Used for densitometric quantification** |   **Ignore the last line**  **Used for densitometric quantification** |  |
| **N1** | **N2** | **N3** | **ICP8** |
|   **SELECTED FOR FIGURE** |  |   **Ignore the last line (H4 cells 24 hpi)** |  |
| **N1** | **N2** | **N3** | **ß-ACTIN** |
|   **Ignore the last line is a sample from another set**  **Used for densitometric quantification**  **SELECTED FOR FIGURE** |   **Used for densitometric quantification** |   **Used for densitometric quantification** |  |

| **FIGURA 5.H**  **HaCaT cells** |  | | |
| --- | --- | --- | --- |
| **N1** | **N2** | **N3** | **KLK6 SUPERNATANT** |
|   **First one order of lines: 2,1,3,4,5,6** |   **Second one, last line ripa pellet 18hpi** |   **Last line ripa pellet 18hpi**  **SELECTED FOR FIGURE** |  |
| **N1** | **N2** | **KLK6 PELLET** | |
|   **SELECTED FOR FIGURE** |  |  |  |
| **N1** | **N2** | **ICP8** | |
| **SELECTED FOR FIGURE** |  |  |  |
| **N1** | **N2** | **ACTIN** | |
|   **SELECTED FOR FIGURE** |   **Ignore the last line** |  |  |
| **FIGURA 6.A**  **HaCaT cells** |  | | |

| **N1** | **N2** | **N3** | **KLK6** |
| --- | --- | --- | --- |
|   **Order of Lines 1-10:**  **C/HSV1 – S/HSV1 – C/HSV1-CHX (3-6) – S/HSV1 C/CHX (7-10)**  **Used for densitometric quantification** |   **Used for densitometric quantification** |   **Used for densitometric quantification**  **SELECTED FOR FIGURE** |  |
| **N1** | **N2** | **N3** | **ICP8** |
|   **Order of Lines 1-10:**  **C/HSV1 – S/HSV1 – C/HSV1-CHX (3-6) – S/HSV1 C/CHX (7-10)** |   **SELECTED FOR FIGURE** |  |  |
| **N1** | **N2** | **N3** | **ß-ACTIN** |
|   **Order of Lines 1-10:**  **C/HSV1 – S/HSV1 – C/HSV1-CHX (3-6) – S/HSV1 C/CHX (7-10)**  **Used for densitometric quantification** |   **Used for densitometric quantification** |   **Used for densitometric quantification**  **SELECTED FOR FIGURE** |  |
|  | | | **KLK6 HIGH EXPOSURE** |

| **FIGURA 6.C**  **HaCaT cells** |  | | | | |
| --- | --- | --- | --- | --- | --- |
| **N1** | **N2** | **N3** | **N4** | **N5** | **KLK6** |
|   **Used for densitometric quantification** |   **Used for densitometric quantification** |   **Used for densitometric quantification** |   **Used for densitometric quantification** | **Used for densitometric quantification** |  |
| **N1 (Left), N2 (right)**  **N1 SELECTED FOR FIGURE** | | | | | **KLK6** |
| **N1** | **N2** | **N3** | **N4** | **N5** | **ICP8** |
| **SELECTED FOR FIGURE** |  |  |  |  |  |
| **N1** | **N2** | **N3** | **N4** | **N5** | **ß-ACTIN** |
| **Used for densitometric quantification**  **SELECTED FOR FIGURE** | **Used for densitometric quantification** | **Used for densitometric quantification** | **Used for densitometric quantification** | **Used for densitometric quantification** |  |
|  |  |  |  |  |  |

| **FIGURA 6.E**  **HaCaT cells** |  | | |
| --- | --- | --- | --- |
| **N1** | **N2** | **N3** | **KLK6** |
| **Used for densitometric quantification** | **Used for densitometric quantification**  **SELECTED FOR FIGURE** | **Used for densitometric quantification** |  |
| **N1** | **N2** | **N3** | **ICP8** |
|  | **SELECTED FOR FIGURE** |  |  |
| **N1** | **N2** | **N3** | **ß-ACTIN** |
| **Used for densitometric quantification**  **SELECTED FOR FIGURE** | **Used for densitometric quantification** | **Used for densitometric quantification** |  |

| **FIGURE 7**  **HaCaT cells** |  | | |
| --- | --- | --- | --- |
| **N1** | **N2** | **N3** | **KLK6** |
| **Used for densitometric quantification**  **SELECTED FOR FIGURE** | **Used for densitometric quantification** | **Used for densitometric quantification** |  |
| **N1** | **N2** | **N3** | **ICP27** |
|  |  | **SELECTED FOR FIGURE** |  |
| **N1** | **N2** | **N3** | **ICP0** |
|  | **SELECTED FOR FIGURE** |  |  |
| **N1** | **N2** | **N3** | **ICP8** |
|  | **SELECTED FOR FIGURE** |  |  |
| **N1** | **N2** | **N3** | **ICP5** |
|  | **SELECTED FOR FIGURE** |  |  |
| **N1** | **N2** | **N3** | **ß-ACTIN** |
| **SELECTED FOR FIGURE** |  |  |  |
| **N1** | **N2** | **N3** | **HSV1 TOTAL** |
|  | **SELECTED FOR FIGURE** |  |  |

| **SUPPLEMENTARY FIGURE 4.A**  **HaCaT cells** |  | | |
| --- | --- | --- | --- |
| **N1** | **N2** | **N4** | **KLK1** |
| **Used for densitometric quantification**  **SELECTED FOR FIGURE** | **Used for densitometric quantification** | **Used for densitometric quantification** |  |
| **N1** | **N2** | **N5** | **KLK12** |
| **Used for densitometric quantification** | **Used for densitometric quantification** | **Used for densitometric quantification**  **SELECTED FOR FIGURE** |  |
| **N1** | **N2** | **N5** | **ß-ACTIN** |
| **Same protein set than**  **FIGURE 5.A and 5F** | **Same protein set than**  **FIGURE 5.A and 5F** | **SELECTED FOR FIGURE** |  |

| **SUPPLEMENTARY FIGURE 4.D**  **HaCaT cells** |  | | |
| --- | --- | --- | --- |
| **N1** | **N3** | **N4** | **KLK5** |
| **Used for densitometric quantification** | **Used for densitometric quantification** | **Used for densitometric quantification**  **SELECTED FOR FIGURE** |  |
| **N2** | **N4** | **N5** | **KLK7** |
| **Used for densitometric quantification** | **Used for densitometric quantification** | **Used for densitometric quantification**  **SELECTED FOR FIGURE** |  |
| **N1** | **N3** | **N4** | **KLK10** |
| **Used for densitometric quantification** | **Used for densitometric quantification**  **SELECTED FOR FIGURE** | **Used for densitometric quantification** |  |
| **N1** | **N3** | **N4** | **ß-ACTIN** |
| **Same protein set than**  **FIGURES 5.A and 5F** | **Ignore the last line (H4 cells 24 hpi)**  **Used for densitometric quantification**  **Same protein set than**  **FIGURES 5.A and 5F**  **SELECTED FOR FIGURE** |  |  |

| **SUPPLEMENTARY FIGURE 4.H**  **HaCaT cells** |  | | | |
| --- | --- | --- | --- | --- |
| **N1** | **N4** | **N6** | | **KRT10** |
| **Used for densitometric quantification**  **SELECTED FOR FIGURE** | **Used for densitometric quantification** | **Used for densitometric quantification** | |  |
| **N3** | **N4** | **N5** | | **P53** |
| **Used for densitometric quantification** | **Used for densitometric quantification**  **SELECTED FOR FIGURE** | **Used for densitometric quantification** | |  |
| **N3** | **N4** | **N5** | **N6** | **ß-ACTIN** |
| **Same protein set than**  **FIGURES 5.A and 5F**  **SUPPL. FIGURE 4D** | **Same protein set than**  **SUPPL. FIGURES 4.A and 4D**  **SELECTED FOR FIGURE** | **Same protein set than SUPPL. FIGURES 4A and 4D** |  |  |
